# Cost-Constrained feature selection in binary classification: adaptations for greedy forward selection and genetic algorithms

**DOI:** 10.1186/s12859-020-3361-9

**Published:** 2020-01-28

**Authors:** Rudolf Jagdhuber, Michel Lang, Arnulf Stenzl, Jochen Neuhaus, Jörg Rahnenführer

**Affiliations:** 1 Department of Statistics, TU Dortmund, Vogelpothsweg 87, Dortmund, 44227 Germany; 2 numares AG, Am BioPark 9, Regensburg, 93053 Germany; 3 Klinik für Urologie, Universitätsklinikum Tübingen, Hoppe-Seyler-Str. 3, Tübingen, 72076 Germany; 4 Universitätsklinikum Leipzig AöR, Department für Operative Medizin, Klinik und Poliklinik für Urologie, Liebigstr. 20, Leipzig, 04103 Germany

**Keywords:** Feature cost, Genetic algorithm, Budget constraint, Cost limit, Feature selection

## Abstract

**Background:**

With modern methods in biotechnology, the search for biomarkers has advanced to a challenging statistical task exploring high dimensional data sets. Feature selection is a widely researched preprocessing step to handle huge numbers of biomarker candidates and has special importance for the analysis of biomedical data. Such data sets often include many input features not related to the diagnostic or therapeutic target variable. A less researched, but also relevant aspect for medical applications are costs of different biomarker candidates. These costs are often financial costs, but can also refer to other aspects, for example the decision between a painful biopsy marker and a simple urine test. In this paper, we propose extensions to two feature selection methods to control the total amount of such costs: greedy forward selection and genetic algorithms. In comprehensive simulation studies of binary classification tasks, we compare the predictive performance, the run-time and the detection rate of relevant features for the new proposed methods and five baseline alternatives to handle budget constraints.

**Results:**

In simulations with a predefined budget constraint, our proposed methods outperform the baseline alternatives, with just minor differences between them. Only in the scenario without an actual budget constraint, our adapted greedy forward selection approach showed a clear drop in performance compared to the other methods. However, introducing a hyperparameter to adapt the benefit-cost trade-off in this method could overcome this weakness.

**Conclusions:**

In feature cost scenarios, where a total budget has to be met, common feature selection algorithms are often not suitable to identify well performing subsets for a modelling task. Adaptations of these algorithms such as the ones proposed in this paper can help to tackle this problem.

## Background

Feature selection is an important and widely applied preprocessing step in the field of biomarker detection. In high-dimensional data sets, which are often found in the “-omics” field (genomics, transcriptomics, proteomics, metabolomics), many input variables may not carry relevant information for a given task. Others may represent redundant information. Excluding these features from the model building process can drastically improve predictive power in such situations.

Often the selection of a suitable feature subset is not driven solely by performance issues. Costs for the inclusion of certain features can be another aspect to consider. These costs may not only refer to financial aspects, but can be seen as a general construct to take into account any disfavoured aspect of a feature. This could represent a time span to raise a feature, a failure rate of the measuring process, or the patient harm during the sample taking process. Incorporating costs follows the idea of limiting the total model cost, which we call budget in this paper. If this budget is flexible and can be adapted if necessary, we refer to a *soft margin* budget. Soft margin budgets have been investigated in the context of feature selection under the name *cost-sensitive learning* [1, 2]. This field covers flexible approaches harmonizing costs of misclassification and costs of features [3]. Approaches of cost-sensitive learning may be useful for situations, where the goal is a trade-off between predictive performance and costs.

If a budget refers to a fixed limit, we call this situation a *hard-margin* budget. For a given feature selection problem it can be seen as an additional constraint. In practice, a hard-margin budget could be e.g. available money, available time, or official regulations on test failures. Initial research on the hard-margin situation was presented by Min et al. [4] who introduce a thorough problem definition in the context of rough sets and present feature selection heuristics. Extensions of this work can be found in Min et al. [5]. An implementation of a simple genetic algorithm with a cost-constrained fitness function is introduced in Liu et al. [6].

We aim to broaden this field of research by proposing multiple cost-constrained extensions to well known feature selection algorithms. We adapted a standard greedy forward feature selection to handle cost constraints. A similar adaptation, but in the context of submodular performance functions, was proposed by Leskovec et al. [7]. They generalize a greedy algorithm by dividing the gain in performance of a feature by the additional cost of this feature. They compute two solutions, one using such a cost adaptation and one with uniform costs, and select the solution with better performance. They prove that the resulting performance is at least a constant fraction (≈63*%*) of the optimal solution [7]. In our approach we select features by a general performance measure that is typically not submodular. We also consider both, cost-adapted and unadapted, methods separately, instead of the described hybrid approach, such that theoretical error limits cannot be transferred. However, we provide a practical and computationally more efficient method.

Besides the greedy approach, we implemented two adaptation strategies for genetic algorithms [8]. As baseline approaches for all of these methods we implemented an unadapted greedy forward feature selection and a simple cost-constraining strategy for four filter methods [9] suggested in Bommert et al. [10].

We evaluated all methods in 11 artificial simulation settings and two simulation studies based on real-world data sets. As quality measures we consider predictive performance, run-time, detection rate of relevant features, and model size. Each data setting is designed to mimic typical real-world scenarios. Feature costs are generated randomly and different extents of budget constraints are analyzed.

The paper starts with a thorough description of the proposed methods. The third section introduces the design of the simulation studies. Multiple settings on artificial data and two real-world data sets are considered to evaluate the methods. The results of these simulations are summarized in the fourth section. In the final section we discuss our findings, give recommendations on the application of the methods and suggest further extensions to this work.

## Methods

### Problem definition

Given a data set *D*, the goal of feature selection is to find a feature combination (feature set) *s* within the power set $\mathcal {P}(\{X_{1},\dots,X_{p}\})$ of all features *X*_1_,…,*X*_*p*_ for which a statistical model *M*(*s*|*D*) is optimal with respect to a performance criterion *Q*. Assume that the optimal value of the performance criterion is the minimal value. Concisely, the problem can then be formulated as

1 $$ \hat{s} = \underset{s}{\arg{\min}}\left\{Q(M(s|D))\right\}  $$

In many real-world scenarios, obtaining a feature *X*_*i*_ may cause individual additive feature costs *c*_*i*_. A fixed cost budget *c*_max_ for the feature combination *s* on the one hand decreases the complexity of the problem by reducing the number of possible candidate solutions, but on the other hand complicates the search strategy by introducing an additional constraint. This constraint can be defined as

2 $$ \sum_{i:X_{i}\in s} c_{i} \le c_{\max}.  $$

Many algorithms have been developed to efficiently solve the standard feature selection problem. For cost-constrained data situations, filter method results can be constrained post hoc. We implemented one such strategy as a baseline method for comparisons. However, for more sophisticated feature combination search algorithms more elaborated specific changes are required to adapt them to cost-constrained settings. In the following, we introduce several such methods.

### Adaptations of greedy forward selection

#### Forward selection with naive cost limitation (FS)

Greedy forward selection is a popular technique for feature subset selection. The main advantage of this approach is its simplicity and generally low run-time in small feature spaces. This makes greedy forward selection applicable to many practical problems. The algorithm starts with an empty set *s*_0_. Then, iteratively the currently optimal additional feature *X*_*i*_ with respect to a performance measure is added to the set. A typical choice for this measure is the Akaike Information Criterion (AIC) [11]. Minimizing the AIC can be interpreted as an optimization of the trade-off between goodness of fit and model size. By not solely focusing on goodness of fit, the AIC also defines an implicit stopping criterion. If no sufficient performance improvement can be achieved to justify adding an additional feature the current solution is returned.

In the context of a limited feature cost budget, however, this approach in general does not guarantee an admissible solution. A trivial adaptation to ensure that the constraint is fulfilled would be to simply redefine the stopping criterion. If a budget violation is detected after adding a feature, the algorithm is terminated and the prior solution is returned. This may lead to premature stopping at any point, where another useful candidate with lower cost could still be added.

A superior alternative is to subset the candidates in every iteration to only include those, which will not exceed the budget if added. This simple forward selection adaptation is labeled FS in this paper and is implemented as a naïve approach as a comparison in the simulation studies. As FS does not actually weigh costs in the selection process, but only limits the overall budget, it can seen as a baseline for the proposed adaptations of the greedy forward selection described in the following sections.

#### Cost constraint forward selection (cFS)

In the presence of feature costs, the suitability of a single feature candidate does not only depend on its contribution to model performance, but also on its relative cost. A feature, which is ten times cheaper compared to another one, but performs almost identically, is intuitively a clearly better choice to be selected. To formulate this intuition, we introduce the Benefit-Cost Ratio (BCR). Using the AIC as measure for performance, the cFS of adding a feature *X*_*i*_ to a candidate set *s* is given by

3 $$ \text{BCR} = \frac{\text{AIC}(M(s|D)) - \text{AIC}(M(s\cup X_{i}|D))}{c_{i}}  $$

This measure quantifies the gain in performance due to a feature relative to its additional costs. We adapt the FS algorithm by iteratively adding the candidate feature resulting in the highest cFS. One advantage of this idea is that in scenarios with highly correlated data the selection of cheaper surrogate features with similar information is encouraged. The stopping criterion for this algorithm does not need to be adapted, since

$$\text{AIC}(M(s|D))\le\text{AIC}(M(s\cup X_{i}|D)\iff\text{BCR}\le 0.$$

As an uninformative feature should never be added even if it is cheap, it is sufficient to stop if no improvement in performance can be achieved. The implementation of the proposed *cost-constrained forward selection* (cFS) is given in Algorithm 1.


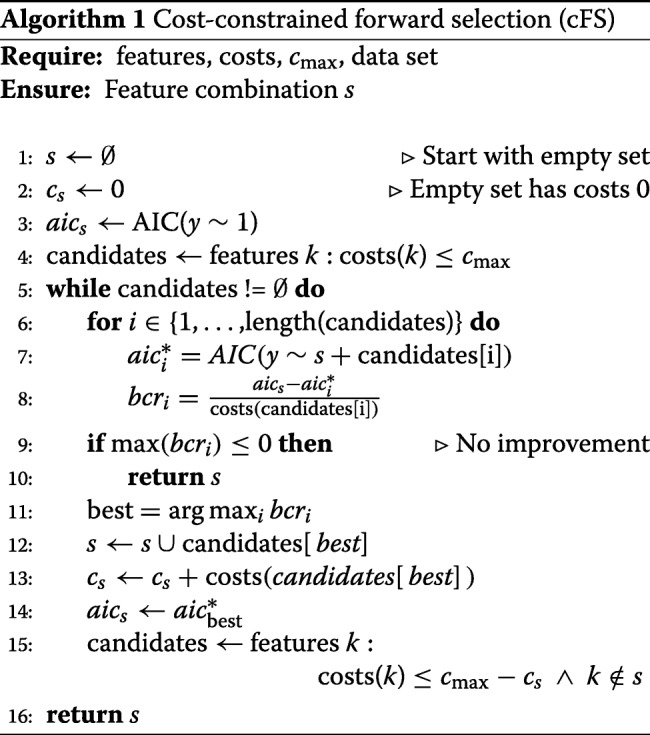


How to deal with the trade-off between benefit and cost plays an essential role for the outcome of the feature selection. The FS algorithm on the one hand solely assesses the benefit of a feature and ignores costs until the budget limits the available feature pool. In contrast, the cFS algorithm values cost equally high as benefit in predictive performance, which might be too rigorous in some applications. A trade-off between these two extrema can be formulated by adapting the cFS definition:

4 $$ \text{BCR}_{\xi} = \frac{\text{AIC}(M(s|D)) - \text{AIC}(M(s\cup X_{i}|D))}{c_{i} + \xi}  $$

We propose two choices for *ξ*, which are thoroughly evaluated in the simulations of this paper. The first option is $\xi = \underset {i\in \{1,\ldots,p\}}\max (c_{i})$. The corresponding feature selection method is called cFS.max. With this adaptation, the focus of the cFS is strongly altered towards the benefit in AIC. A more moderate version is given by $\xi = \frac 1p\sum _{i=1}^{p} c_{i}$, and the corresponding algorithm is called cFS.mean. In real-world applications it is important to first assess the cost distribution and then adapt these measures if necessary. For example, mean and maximum can be prone to outliers with very high costs. Respective definitions of *ξ* using quantiles may be more suited in such situations.

### Adaptations on genetic algorithms

#### Genetic algorithm without cost constraints

Genetic algorithms are heuristic search algorithms based on the evolutionary ideas of survival of the fittest, genetic crossover and random mutation. They were first introduced by Holland [8]. Genetic algorithms are known to be well suited for combinatorial problems and hence are often used for feature selection in machine learning applications. The base algorithm starts by generating an initial population of candidate feature combinations. Each of these combinations is evaluated using a so-called *fitness function*, which assigns a real value to the combination. In applications of feature selection this value can represent a measure of model performance. The results of the fitness evaluation are the basis for the *genetic operators*, which are used to suggest a new candidate population for the next generation. Genetic operators can be subdivided into three groups:

- Selection operators
- Crossover operators
- Mutation operators

Selection operators decide, which elements of the current population will proceed to the next generation. Decisions are based on the current fitness evaluations. A practical example is *lrSelection*, which is implemented in the R package GA [12]. This selection operator assigns a probability of proceeding to the next generation based on the fitness rank in the current population. The evolutionary idea of survival of the fittest corresponds to this operator.

Crossover operators can be seen as a recomposition of two feature combinations (’parents’) into two new combinations (’children’). These children may comprise elements of both parents. An implementation for this idea is *uCrossover* (R package GA [12]). This function transfers all features present in both parents to the children and makes a coin-toss for every other feature present in only one parent to decide if it will be carried over to a child or not. The corresponding evolutionary idea of the crossover operator is genetic crossover.

Mutation operators randomly alter feature combinations to further explore unknown regions of the feature space. This alteration can mean to remove a random feature from the model, or to add a random new feature to it. An implementation for this idea is *raMutation* (R package GA [12]). This function randomly chooses a feature from the total feature pool. If the chosen feature is part of the current feature set, it is removed, and if not, it is added to it. The mutation operator is meant to resemble the evolutionary idea of genetic mutation.

Typically, a user does not need to implement genetic operators from scratch. Besides the mentioned implementations many more pre-defined algorithms exist to choose from [12]. The generation of a new population using the genetic operators and the fitness evaluation of this population are iterated until a pre-defined convergence criterion is fulfilled. When not considering restrictions on feature costs this approach can result in budget violations. In the following sections we introduce two strategies to alter the genetic algorithm to handle budget constraints.

#### Genetic algorithm with fitness function adaptation (fGA)

The central element of a genetic algorithm is the fitness function. It assigns a real number assessing the suitability of a candidate set *s*. For candidate sets that violate the budget the fitness value may be set to a constant negative value indicating an unsuited feature combination. This is for example done in the implementation of Liu et al. [6]. The downside of this approach however is that no information can be gained from the constraint violations. The algorithm may need to evaluate a large number of models before finding a first valid candidate set.

**Fig. 1:**
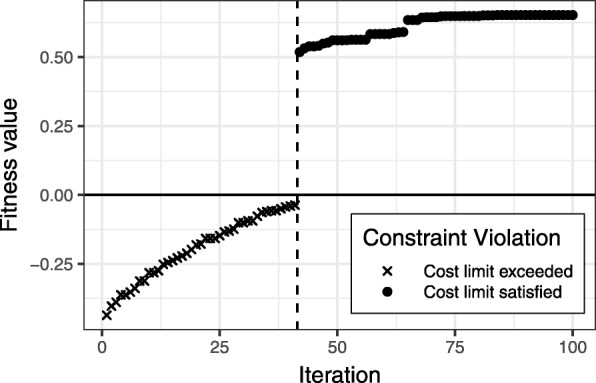
Optimization path of a (not cost-adapted) genetic algorithm that uses a fitness function accounting for the extent of constraint violation. Data: 298 features (each with cost 1), *c*_max_=10. The first candidate meeting the constraint is found in iteration 42

An alternative is to specify the extent of constraint violation in the fitness function. This way, the genetic algorithm is able to evolve from higher constraint violations to lower ones, eventually finding valid candidate sets. Figure 1 shows an optimization path of a genetic algorithm, where the fitness value of a candidate set is defined by

5 $$ \text{fitness}(s) = \left\{ \begin{array}{lr} 1-\frac{costs(s)}{c_{\max}}, & \text{if costs} > c_{\max}\\~\\ \text{AIC}(M(s|D))^{-1}, & \text{if costs} \le c_{\max} \end{array}\right..  $$

In the example in Fig. 1, the algorithm is able to approach the constraint region with each step, eventually finding a first suitable candidate in iteration 42. For comparison, an implementation of a constant violation term of 0 (with random initial population) did not find a single valid candidate in the exact same scenario after 1000 iterations.

Besides the fitness function, the initial population plays an important role for the convergence rate of the algorithm. Starting within or close to the constraint region can spare many iterations of evaluating non-suitable candidates. Random feature combinations with an average total cost of *c*_max_ can be drawn by letting each of the *p* features have a probability of $\min (1,c_{\max }/\sum _{i=1}^{p} c_{i})$ to be part of a candidate set.

Using this initialization and the flexible constraint violation term of (5), we propose the *genetic algorithm with fitness adaptation* (fGA). For comparability with the cFS algorithm, the fitness measure for candidate sets within the budget is based on the AIC. As the fitness needs to be maximized and negative values are reserved for constraint violations, AIC(*M*(*s*|*D*))^−1^ is used in the implementation. For the genetic operators, the earlier mentioned pre-defined methods *lrSelection*, *uCrossover* and *raMutation* of the R package GA [12, 13] are typical choices in feature selection applications and are here used for the algorithm fGA.

#### Cost-preserving genetic algorithm (cGA)

When using the fGA, a lot of models that do not meet the budget constraint may be proposed at any point. A different approach to this problem is to alter the search path in a way that only valid models enter the evaluation population at any time. This way, an adaptation of the fitness function becomes obsolete and the total amount of fitness evaluations can be reduced. Reducing fitness evaluations can be particularly relevant for situations with computationally expensive fitness functions. To realize this approach, all genetic operators that can lead to constraint-violating candidates need to be adapted. These are crossover and mutation. Additionally, the initial population needs to be chosen more strictly to avoid choosing sets with violations. The practical implementation of cost-optimized versions for the genetic operators and the initial population generation is described in the following sections.

##### Cost-optimized population initialization

As already mentioned in the previous section, the definition of an initial population plays an important role for the convergence time of a genetic algorithm with fitness adaptation. It is also the first possible source of constraint violations. The simple initial population algorithm used for the fGA drastically reduces this problem, it however does not ensure that every candidate set is within the budget. A suitable function needs to create a broad variety of different feature combinations solely in the subspace defined by the budget constraint. For this purpose, we propose a random forward selection approach, which is described in Algorithm 2.


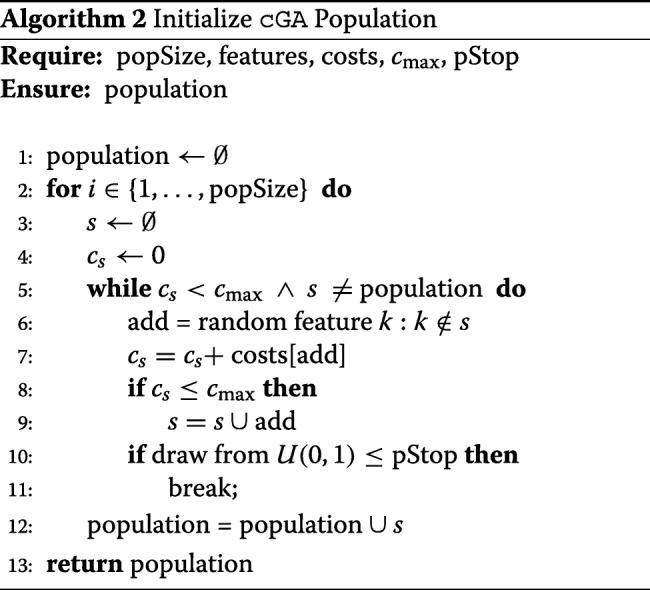


Algorithm 2 creates a candidate feature combination by starting with an empty set and subsequently adding random features until the budget is exceeded. At every step, the current set can be returned with a user defined probability pStop. This way, any feature combination within the budget has a chance of entering the initial population. In the similar cFS algorithm (Algorithm 1) we restricted the random draws to features with costs smaller than the remaining budget of the current feature combination. This is not done here. Hence, the feature combinations are not pushed towards perfectly reaching the cost limit, which would not necessarily give an advantage here but would favor the selection of very cheap features. In the practical applications of this paper, pStop is set to $\frac 1{500}$. With this rather small choice, the initial population will preferably consist of large candidate sets.

##### Cost-optimized selection operator

As this procedure is not affected by the presence of individual feature costs, an adaptation of this genetic operator is not necessary. In the practical applications of this paper, the *lrSelection* of the R package GA [12, 13] is used.

##### Cost-optimized crossover operator

In the presence of cost limits, a standard crossover approach can lead to various problems. Assume a pool of 500 features, each having a cost of 1, and a total cost limit of *c*_max_=10, with the number of informative features being much greater than 10. After a number of generations, parent combinations may already fill the budget quite well. A crossover of two parents with 10 different of the 500 features will create a cost constraint violation in more than 40% of cases.^1^

By adapting the crossover function with respect to costs, these violations can be omitted. The proposed cost-constrained crossover algorithm is given in Algorithm 3.


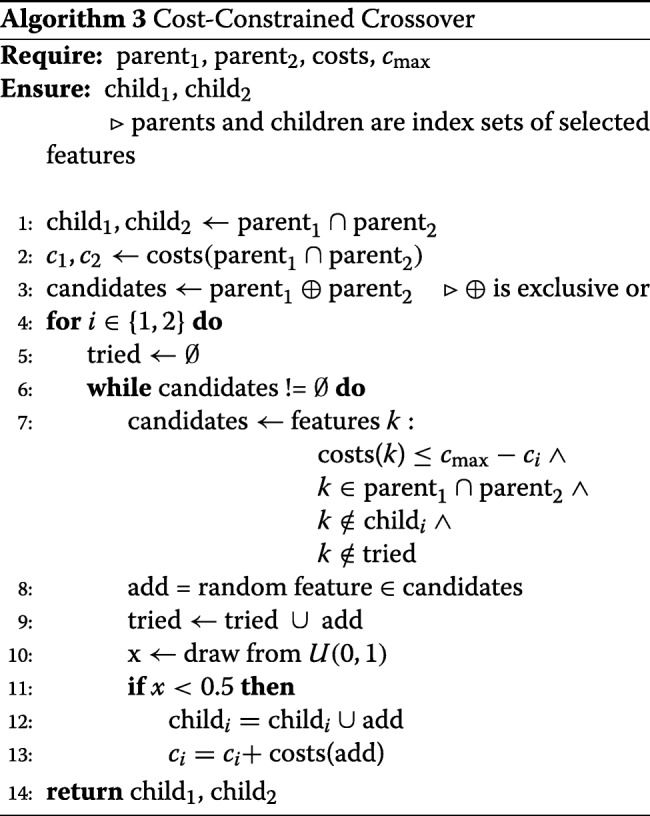


The algorithm starts by forwarding features present in both parents to the children, as those are preserved in any crossover scenario. Possible candidates to be added are only those, which are present in exactly one parent, are not exceeding the remaining budget and have not been selected in the current crossover run before. A random candidate is drawn and added to the child set with probability 0.5 (i.e. each parent has the same chance of transcribing its information onto the child). The algorithm stops after either every candidate has been tried, or the budget does not allow any further candidate to be added.

##### Cost-optimized mutation operator

The final genetic operator that can result in budget violations is the mutation operator. This operator adds or removes one random feature from the current candidate set. While selection and crossover mainly exploit regions of the feature space that are already known to perform well, the idea of mutation is to further explore random feature alterations. This can help to escape from local optima. In the context of a cost limit, however, the downside of a random mutation is that carelessly adding a feature may exceed the budget. Recall the example scenario presented in the previous section with 500 features and cost limit 10. There, after some iterations, most feature sets may exhaust the budget completely. Then, a random mutation will add another feature in 98% of cases^2^ and hence violate the budget.

To overcome this problem, we propose an adaptation of the mutation operator that avoids solutions outside the budget. In the cost-agnostic version, first a random feature is chosen. If this feature is already a member of the current set, then it is removed, otherwise it is added. Hence the decision to remove or add is implicitly modeled by the random selection of a feature. To guarantee a solution inside the budget, our mutation operator models this decision explicitly. At each step, we decide in advance if we add a feature to the current set *s*, or if we remove one. The probability for this decision is

6 $$ \mathbb{P}(\mathrm{Add~a~feature}) = 1 - \frac{\sum_{i:X_{i}\in s}c_{i}}{c_{\max}}  $$

The idea is to add a feature with higher probability if the total cost of the current set is still far away from the budget limit, and otherwise to rather remove a feature to better explore the search space. The decision which feature actually is added or removed is made in a second step by choosing randomly from all possible features. In the extreme case that in the first step the decision is to add a feature, but none meets the budget constraint, instead a random feature is removed. The proposed mutation strategy is given in Algorithm 4.


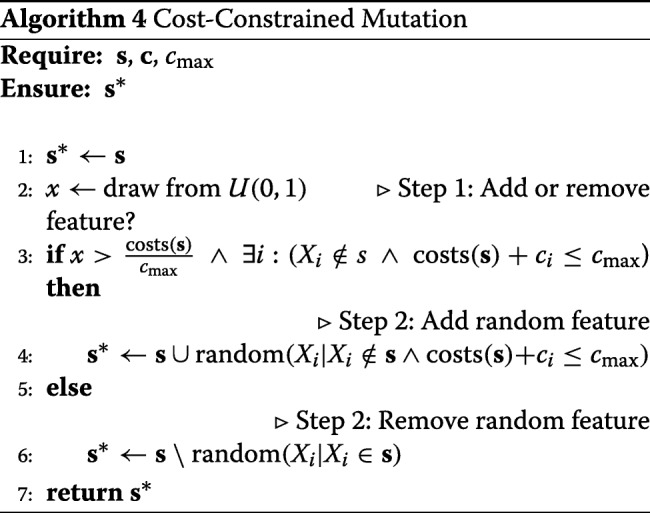


An alternative implementation of the mutation operator could be formulated as follows: We only consider features, which if altered will not exceed the cost budget. This includes every candidate that is already part of the current feature set. The chance of adding a candidate is thus implicitly modeled by the ratio of possible candidates and current model size.

Both ideas lead to valid results for the given problem. For the applications in this paper the two step approach described in Algorithm 4 is applied.

##### Overall implementation of cGA

This section summarizes the proposed cost-preserving genetic algorithm. The initial population is generated by a random forward selection (Algorithm 2). For the genetic operators *lrSelection* (R package GA [12, 13]) is used for selection, *cost-constrained crossover* (Algorithm 3) is used for crossover and *cost-constrained mutation* (Algorithm 4) is used for mutation. As the fitness function does not need to handle constraint violations, any measure of performance can be used without adaptation. For comparability with the other proposed methods, the negative AIC was chosen in the simulations of this paper.

### Implementation of baseline filter methods

Besides the proposed methods of the previous sections, multiple filter methods are implemented as baseline approaches for the simulations. Filters compute a measure of importance for every feature that in a second step can be used to select a suitable feature subset. Filter methods are simple to use and can easily be adapted to handle cost constraints. A popular filter choice for binary response variables is based on the t-test. For every candidate feature, the p-value of a two-sample t-test for the groups defined by the response variable is computed. This value is then used as a measure for the discriminative power of the respective feature. Further filter methods implemented in this paper were chosen according to the recommendations of a recent benchmark study by Bommert et al. [10]. They compared 22 filter methods on 16 high-dimensional data sets with respect to predictive performance and run-time. The methods Filter.Symuncert, Filter.PraznikJMIM and Filter.RangerImpurity showed a good compromise between both aspects. The former two methods use an entropy based feature evaluation, while the latter one assesses the node impurity of random forests. For further information on these methods see Brown et al. [14], Izenmann et al. [15], or Bommert et al. [10]. All analyzed filter methods are used with their implementation within the R package mlr [16].

To select a final model from the feature ranking of the filter methods, which also meets a given budget constraint, a top-down approach is used. Features are added to the model in order of their rank according to the filter, but only, if the cost of the resulting model does not exceed the budget. The process is stopped either, if the cost of any remaining feature would exceed the budget or, if a certain threshold set for the feature importance measure is reached. However, the latter option should be handled with care as the values for feature importance often represent abstract measures without a natural limit regarding usefulness. For the simulations, the threshold for Filter.tTest is set to 0.05 and for Filter.Symuncert it is set to 10^−6^. For the remaining methods no threshold is set.

## Simulation studies

### Artificial data settings

The goal of our simulation studies is to thoroughly evaluate the characteristics of the proposed methods and to compare them to plausible alternatives in the context of a limited feature budget. We consider binary classification problems, i.e. a binary response variable and *p* variables emerging from a *p*-dimensional multivariate normal distribution. In the simulation studies we vary the total number of independent variables *p*, the number of truly relevant features *p*^(rel)^≤*p*, the effect size *β* and the feature cost scenario. For each main setting, the cost of the *i*-th feature *c*_*i*_ is drawn from a uniform distribution $\mathcal {U}(0.1,1)$. We therefore implicitly assume a situation, where the cost of a feature is independent of its effect size. As an extension, we analyze additional scenarios that also consider effect dependent costs (see “Settings with altered simulation design” section).

We define the budget *c*_max_ relative to the total cost of all relevant features via a parameter *γ*≥0. A value of *γ*=1 corresponds to a budget, where all relevant features exactly fit in. For *γ*≤1 the value *c*_max_ is the sum of the cheapest costs up to the *γ*-quantile of the empirical cost distribution of all relevant features. For example, for *γ*=0.5 this means that at maximum only the cheapest 50% of the relevant features can be added. *γ*>1 represents the situation, where the budget is greater than the sum of the costs of all relevant features. A total of *γ*·*p*^(rel)^ average features can be added here. To compute *c*_max_ the cost of an additional noise feature is considered as the overall average feature cost $\bar {c}$. For example a value of *γ*=1.5 allows the inclusion of all *p*^(rel)^ relevant features together with 0.5·*p*^(rel)^ additional noise features with average cost $\bar {c}$. Introducing the parameter *γ* allows to define the cost constraint in a relative manner by the proportion of relevant information that is allowed. Altogether this can be formulated as

7 $$ c_{\max} := \left\{ \begin{array}{lr} \sum_{i:\beta_{i}>0} c_{i} \cdot I(c_{i}\le q_{\gamma}^{(\text{rel})}), & \text{if }\gamma \le 1\\~\\ \sum_{i:\beta_{i}>0} c_{i} + (\gamma - 1)\cdot \bar{c}\cdot p^{(\text{rel})}, & \text{if }\gamma > 1 \end{array},\right.  $$

where *I* is the indicator function, $q_{\gamma }^{(\text {rel})}$ is the *γ*-quantile of the empirical cost distribution of truly relevant features and $\bar {c}$ is the mean cost of all features.

For all main simulation settings, *B*=100 data sets of size *n*=500 are generated as follows (following the simulation framework of Boulesteix et al. [17]). In a first step, the binary response variable is drawn from the Bernoulli distribution $\mathcal {B}(0.5)$. Subsequently, the features are drawn from *p*-dimensional multivariate normal distributions

8 $$\begin{array}{*{20}l} X_{1}, \dots, X_{p} | Y=1 &\sim \mathcal{N}_{p}(\boldsymbol\mu, \boldsymbol\Sigma),\\ X_{1}, \dots, X_{p} | Y=0 &\sim \mathcal{N}_{p}(\mathbf{0}_{p}, \boldsymbol\Sigma), \end{array} $$

with mean vector ***μ*** defined as

9 $$ \boldsymbol\mu^{T} = \left(\underbrace{\beta,\dots,\beta}_{p^{(\text{rel})}}, \underbrace{0,\dots,0}_{p-p^{(\text{rel})}}\right)  $$

In all settings simulated according to this method, covariance matrices ***Σ*** are chosen to be *p*-dimensional identity matrices **I**_*p*_. To also extend our analyses to a non-independent feature space, a further simulation Setting G is added.

We consider six main settings labeled *A* to *F* for the data parameters *γ*, *p*, *p*^(rel)^ and *β*. Additionally, five settings were generated to analyze more specific scenarios. These scenarios consider a non-identity covariance structure (Setting G), an effect-dependent cost distribution (Setting H), non-constant effect sizes for relevant parameters (Settings I and J) and features that are not normally distributed (Setting K). An overview of the exact parameter configurations for all settings is given in Table 1.

**Table 1:** Combinations of *γ*, *p*, *p*^(rel)^ and *β* used for the simulation design

	*γ*	*p*	*p*^(rel)^	*β*
Setting A	$\frac {1}{2}$	30	18	0.3
Setting B	$\frac {2}{3}$	30	3	1
Setting C	$\frac {1}{3}$	300	30	0.5
Setting D	$\frac {2}{3}$	300	3	0.5
Setting E	2	1500	15	0.5
Setting F	$\frac {1}{2}$	1500	20	0.5
Setting G	$\frac {1}{3}$	300	30	0.3
Setting H	$\frac {1}{3}$	300	30	0.5
Setting I	$\frac {1}{3}$	300	30	$\frac {1}{30}, \frac {2}{30}, \dots, 1$
Setting J	$\frac {1}{3}$	300	30	$\frac {1}{30}, \frac {2}{30}, \dots, 1$
Setting K	$\frac {1}{3}$	300	30	0.5

For every setting *B*=100 training data sets are generated. Settings G to K are specialized settings, which focus on changes in the data generation process. For details see “Settings with altered simulation design” section

Every setting represents a common feature selection task with individual characteristics. We vary the number of input features *p* from only a few (Settings A and B) over several hundreds (Settings C and D) up to over a thousand (Settings E and F). Effect sizes range from 0.3 to 1 standard deviation and the number of truly relevant features ranges from 3 to 30. As the main focus of the simulation concerns situations with budget constraints, *γ* is typically chosen to be small. Yet, we included one setting (Setting E), which does not constraint the relevant features in order to also assess the behaviour of the proposed methods in this situation. A more detailed description of the motivation of the individual settings is given in the results section.

#### Settings with altered simulation design

Five additional settings are introduced where the assumptions of the main simulation design are modified to obtain a more complete picture of the characteristics of the analyzed methods.

##### Setting G

**Fig. 2:**
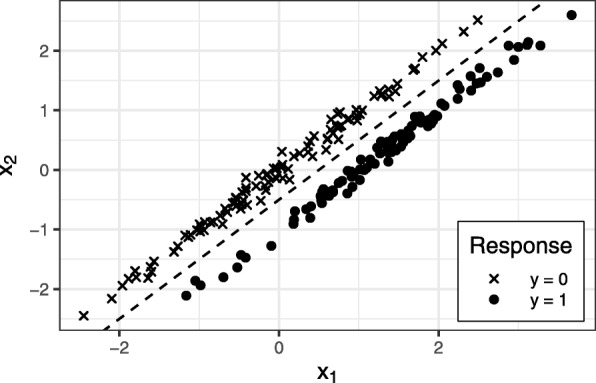
Illustration of a possible situation when applying a non-identity covariance matrix to the situation of (8). Two highly correlated normal distributed features *x*_1_ and *x*_2_, where the first component *x*_1_ has different means for the two classes (*μ*=1 for *y*=1 and *μ*=0 for *y*=0) and the second component *x*_2_ has the same mean (*μ*=0) for both classes. The resulting multivariate structure is perfectly separable by a linear function

In the main design, features are sampled according to (8) with ***Σ*** set to the *p*-dimensional identity matrix **I**_*p*_. However, in most real-world applications, the assumption of completely independent features does not hold true. Metabolomic or genetic data sets often include highly correlated covariables [18]. Since the true processes generating these data are unknown, the covariance matrix of a real metabolomic data set (prostate cancer staging data, see “Real-world data settings” section for further details) was estimated and used to model the dependencies between features. The data generation can be realized in different ways. One option is to insert the estimated covariance matrix as ***Σ*** in the framework of (8). This is a technically valid approach, yet has implications for the resulting multivariate data structure. Figure 2 illustrates an exemplary scenario with strong correlation between a feature *x*_1_ defined as relevant and another feature *x*_2_ with no effect with respect to the binary response *y*.

It can be seen, that these two features create a multivariate structure, which is already perfectly separable by a linear function. With one feature being highly predictive and another highly correlated feature with no predictive power at all, this scenario is on the one hand not resembling a realistic data situation and is on the other hand creating an non-intuitive artificial split between cases and controls.

Therefore, to assess the quality of feature identification, an alternative and more realistic framework is used. We draw the feature matrix from a multivariate normal distribution

10 $$ X_{1}, \dots, X_{p} \sim \mathcal{N}_{p}(\mathbf{0}_{p}, \boldsymbol\Sigma),  $$

where ***Σ*** represents the estimated covariance matrix of the prostate cancer data set. The difference in the number of features (prostate cancer data (298), Setting G (300)) is addressed by appending two additional independent features to the covariance matrix.

We then draw realizations of the response variable *y*_*i*_ from the Bernoulli distribution

11 $$ y_{i} \sim \mathcal{B}(\pi_{i})  $$

with parameter *π*_*i*_ modeled by a logistic relation to the linear predictor of relevant features

12 $$ \pi_{i} = \frac{1}{1+\exp (-\mathbf{x}_{i}^{T}\boldsymbol\mu)}  $$

The structure of ***μ*** is the same as in (9) with value *β* for the first *p*^(rel)^ entries and 0 else. The remaining simulation parameters for Setting G are chosen similar to those in Setting C.

##### Setting H

The second artificial data simulation Setting H addresses the independence assumption of costs and true feature effects. In the main design no information related to costs can be obtained from feature effects and *vice versa*. Therefore it can be seen as the strictest scenario requiring optimization in both directions. However, in practical applications it is plausible to assume that more valuable features might be more expensive on average. To cover this aspect as well, Setting H is introduced, modifying the generation process of the feature costs. Depending on the true effect of *X*_*i*_, the respective costs are drawn from different uniform distributions as follows.

13 $$\begin{array}{*{20}l} \beta_{i}\neq 0 & \;\Rightarrow\; c_{i} \sim \mathcal{U}(0.4, 1)\\ \beta_{i}= 0 & \;\Rightarrow\; c_{i}\sim \mathcal{U}(0.1, 0.7) \end{array} $$

Relevant features have mean cost 0.7, and uninformative features a considerably lower mean cost of 0.4. Besides the cost distribution, all other simulation parameters are adopted from Setting C.

##### Setting I

In all previous settings, the effect sizes of the relevant features are constant. In this case it is clear that the most important features are the relevant ones with smallest cost. To extend our scope, in setting I we consider situations with non-constant effects. We define the true effects sizes of relevant parameters as an equidistant sequence within the interval ]0,1]. The mean vector ***μ*** of definition (9) is changed to

14 $$ \boldsymbol\mu^{T} = \left(\frac{p^{(\text{rel})}}{p^{(\text{rel})}},\dots, \frac2{p^{(\text{rel})}},\frac1{p^{(\text{rel})}}, \underbrace{0,\dots,0}_{p-p^{(\text{rel})}}\right).  $$

This way, we cover a broad and interpretable range from weakly to strongly relevant features. Apart from the definition of ***μ***, all remaining simulation parameters are chosen as in Setting C.

##### Setting J

In Setting H, for constant effects, a dependency between costs and effect size is considered. In the context of non-constant effects, such a dependency may have different implications. Setting J represents a combination of Settings H and I. We use definition (14) for ***μ***, but additionally, we introduce a correlation between *c*_*i*_ and *μ*_*i*_ by drawing costs according to

15 $$ c_{i} \sim \mathcal{U}\left(0.1 + \frac{\mu_{i}}{2}, 0.5 + \frac{\mu_{i}}{2}\right).  $$

For consistency with previous analyses, we again choose all remaining parameters as in Setting C.

##### Setting K

The final additional setting discusses the assumption of normally distributed features. A study by de Torrente et al. [19] with three datasets of the Cancer Genome Atlas with different tumor types [20–22] showed that more than 50% of the genes do not follow a normal distribution. In Setting K, we address this topic by defining a new data generation process similar to Rahnenführer & Futschik [23]. The goal is to draw observations from non-normal distributions with heavy tails. To achieve this, we draw 90% of the observations from a $\mathcal {N}(\mu _{i}, 1)$ and 10% from a $\mathcal {N}(\mu _{i}, 5)$ distribution. The second part adds extreme values to the first main part. This way, we keep effect sizes comparable to other settings, while still generating from a more complex distribution with many possible extreme values. Besides the altered data distribution, the parameters as in Setting C are used.

In each simulation setting, prediction performance for every model is evaluated on an independently drawn test data set of size *n*_Test_=10 000. To compare the analyzed methods, the Area under the Receiver Operating Characteristic Curve (AUC) [24] is computed on this test set. During execution, we measure the run-time of each feature selection task on an AMD Ryzen 7 1700X 3.4 GHz processor with 16GB of RAM. We finally also analyze the number of selected features, and the proportion of relevant features among those. However, in Setting G this measure needs to be interpreted with care as models are not limited to information from the pre-defined relevant features, but can also use correlated surrogate features. For Settings I and J, a simple grouping into relevant and noise features is not reasonable. We provide an alternative analysis on an individual feature level for these settings.

Note that the maximum performance of genetic algorithms also depends on the choice of hyperparameters. We set the population size to 500 and define a maximum number of iterations of 150. Convergence is assumed after 10 consecutive iterations without further improvement. On average, a feature selection with the high dimensional Settings E and F takes around 3 to 5 min with a maximum time-span of over 10 min with this configuration. In practice, depending on the computational resources, one may consider scaling these parameters up for a further increase in performance.

### Real-world data settings

Besides the artificial data simulations described in the previous section, we applied our methods to two real bioinformatics data sets. In the following, we give a short introduction to both settings.

#### Plasmode Setting R

In our first real-world setting, we perform a plasmode simulation study [25]. A so-called plasmode uses a data set generated from natural processes but adds a simulated aspect to the data [26]. For this paper we use a metabolomics data set as basis of our plasmode simulation. From 2013 to 2015, numares AG^3^ received urine samples of prostate cancer patients from cooperation partners of the Universities of Leipzig and Tübingen. Sampling was ethically approved (Leipzig: No. 205-15-01062015, Tübingen: No. 379-2010BO2). Samples were analyzed using nuclear magnetic resonance spectroscopy (NMR). All measurements were carried out on a Bruker Avance II + 600 MHz NMR spectrometer using a PATXI 1H/D-13C/15N Z-GRD probe and a standard pulse program with 30^∘^ excitation pulse and pre-saturation for water suppression (zgpr30). Each rack included one Axinon urine calibrator sample and two Axinon urine controls (numares AG) samples (positioned at the beginning and the end of a rack) in order to assure ideal measurement and reproducibility conditions throughout the run. 1H-NMR spectra underwent automatic data processing and quality control as part of the magnetic group signaling^®^ technology based on spectral properties, such as offset and slope of the baseline in selected spectral regions as well as properties of selected signals, e.g. signal position, shape and width [27].

After processing and quality control 547 samples binned into 298 spectral regions form the basis of our plasmode data set. The integral of the signals in each spectral region is proportional to the abundance of the unknown substances generating the signals. Therefore, these integrals can be used as input features of a predictive model. The so-called binning or bucketing is a typical strategy to create features from an NMR spectrum [28]. Yet, while reducing the data dimension, binning can lead to features with either more than one or no substance at all. In the plasmode context, we use this data set to realistically model the multivariate distribution of the features. To create a controlled scenario, which allows an objective assessment of the analyzed methods, the real relation between features and response variable needs to be known. Hence, the binary response variable is generated from the features that are defined to be relevant (c.f. framework of (8), (9)) To minimize the amount of redundant information, the truly relevant features selected should have minimal covariance. Neighbouring spectral regions could include signals from the same substance, or even only parts of the same signal. To avoid this, a set of relevant features is selected with maximum distance between each other. This way, we observe a median absolute correlation of 0.09 in this setting. The parameter configuration to draw the response variable is chosen similar to Setting C, see Table 2 for exact values.

**Table 2:** Combination of *γ*, *p*, *p*^(rel)^ and *β* used for the plasmode simulation Setting R

	*γ*	*p*	*p*^(rel)^	*β*
Setting R	$\frac {1}{3}$	298	30	0.5

We draw realizations of the response variable *y*_*i*_ from a Bernoulli distribution similar to the approach described in (11) and (12). For the plasmode setting, the structure of ***μ*** is an adapted version of (9) with value *β* at scattered positions for relevant features and 0 everywhere else. However, though all input features are standardized, note that *β*=0.5 cannot be directly compared to the *β* value of the main artificial settings, because the distributions of the features do not follow a normal distribution and contain many extreme outliers. Also, due to the altered response generating process, the expected proportion of values 1 for the target variable is no longer controlled by a fixed hyperparameter and may deviate strongly from 0.5. Here, we obtained a ratio of 0.417 (*y*=1: 228, *y*=0: 319).

As the simulation results of Settings H and J do not suggest a great influence of effect dependent costs, we draw feature costs equivalent to the main settings from the uniform distribution $\mathcal {U}(0.1,1)$. Similarly, the relative definition of *c*_max_ of (7) is used.

Since the data is not split into training and validation set, we generate this split randomly by using approximately $\frac {2}{3}$ of the observations (365) to perform the feature selection and the rest (182) to evaluate the predictive performance of the resulting feature subsets. In our simulation, we consider 100 such random splits and assess the overall results. Similar to the previous section the main focus of the analysis lies on prediction performance, run-time and feature count. An analysis of the proportion of detected true features needs to be assessed with care here as - similar to Setting G - the non-identity covariance of our plasmode data set enables to obtain important predictive information also from originally uninformative features.

#### Setting S

The plasmode simulation of the previous section has the advantage of controlling the relationship between features and response. This allows analyses based on a known truth. But it only provides a partial real-world analysis. For completeness, we also include an analysis on an unmodified real-world data set. Setting S uses the publicly available *Bioresponse data set* from OpenML (ID 4134) [29]. The data set was collected by Boehringer Ingelheim to analyze a biological response of molecules. The binary target variable is positive, if the molecule caused a biological response and negative if not. 1776 features represent molecular descriptors. These are calculated properties that can capture some of the characteristics of the molecule - for example size, shape, or elemental constitution [30].

We simulate costs similarly to Setting R. The main difficulty of Setting S is defining a budget limit. As no information on relevant features is given, we cannot define *c*_max_ as a *γ*-proportion of the total cost of relevant information as in definition (7). We solve this, by roughly estimating the total cost with the computationally fast filter methods. We define a grid of 9 *c*_max_ values between 0 and 4 and compute the performance of the corresponding models selected by the filters. For values above *c*_max_=3 we observe that the performance does not increase any further. An illustration of the results is provided in Additional file 6. We use this value of *c*_max_=3 as our limit in the simulation. To additionally generate a setting with a more notable budget restriction, we also run Setting S with *c*_max_=1.5. By analyzing two limits on a single setting, we can obtain further insight on how results of different methods change when relaxing the budget constraint.

The Bioresponse data contains 3751 observations, which are not split into training and testing set. We follow the same strategy as described in Setting R to generate 100 random data splits. As no underlying truth is known, we only compare the results of the methods, without any reference to an optimal solution. We assess prediction performance, run-time and feature count.

## Results

For each simulation we applied the methods FS (see “Forward selection with naive cost limitation (FS)” section), cFS, cFS.mean, cFS.max (see “Cost constraint forward selection (cFS)” section), fGA (see “Genetic algorithm with fitness function adaptation (fGA)” section), cGA (see “Cost-preserving genetic algorithm (cGA)” section) and the four filter methods Filter.tTest, Filter.Symuncert, Filter.PraznikJMIM and Filter.RangerImpurity (see “Implementation of baseline filter methods” section). However, due to the very similar results compared to their counterparts and for the reasons of clarity we omit the methods cFS.max and fGA in the following sections. Nevertheless, for every result presented, corresponding results including these methods can be found in Additional files 2,3,4 and 5.

### Artificial data simulation results

#### Model performance

**Fig. 3:**
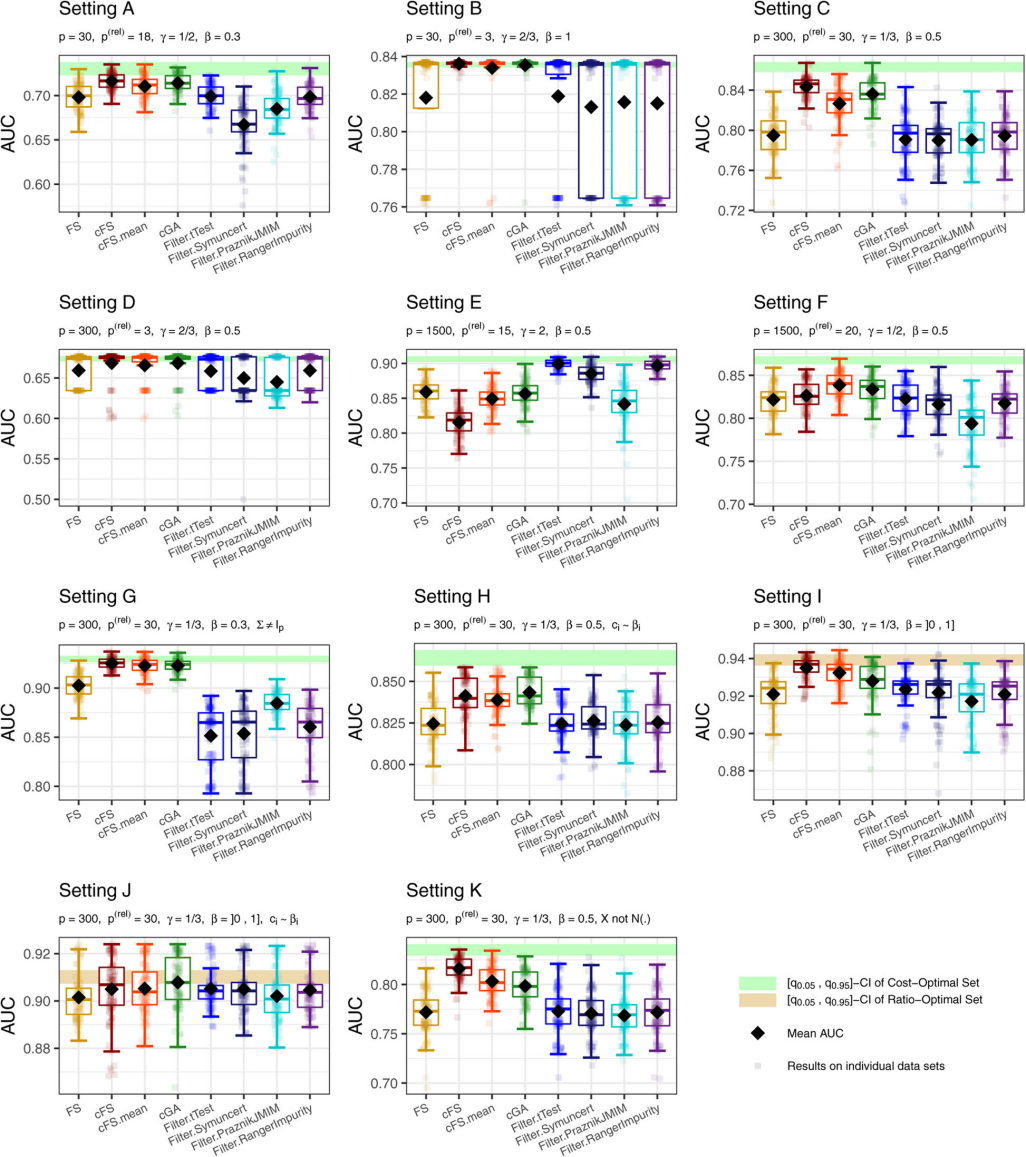
Performance results for simulation Settings A to K. Boxplots for every feature selection method illustrate the distribution of the AUC values obtained in the 100 data sets (transparent dots). The black diamonds depict the mean AUC values. A horizontal bar highlights the area between the 0.05 and 0.95 quantile of AUC values when always selecting the cheapest subset (green) or the best real cFS subset (golden) of relevant features that fit in the budget. Both correspond to a univariately optimal solution

In many applications, the central criterion for a well performing feature selection algorithm is the predictive performance of the resulting model. We assess this aspect using the AUC. Performance results of the analyzed methods for all Settings A to K are shown in Fig. 3. Boxplots are used to illustrate the variation between the 100 simulated data sets. The green bar highlights the 0.05 and 0.95 quantile of the resulting AUC distribution over the simulation data sets when selecting the maximum possible set of features that were defined relevant in the data generation process. In settings with independence between the individual features, this corresponds to the most informative feature combination any feature selection algorithm can achieve. For Settings I and J relevant features are not interchangeable and thus this measure is not applicable. Instead, we provide a golden bar that corresponds to selecting the maximum amount of effect size by selecting features according to their true cFS.

Setting A reflects a situation with a rather small number of candidate features from which 60% carry relevant information. Yet, the cost constraint allows only the cheapest half of the relevant features to enter the model, at best. As there are many informative features to choose from, the challenge for this setting is mainly to find a suitable combination regarding the costs. Here, the proposed methods cFS and cGA show the highest average performance with only slight differences between them. Comparing the forward selection approaches, the cFS selection (using the cFS) results in the highest AUC values. The trade-off adjustment cFS.mean ranks shortly behind, followed by FS. The filter methods show generally lower AUC values. Only Filter.tTest and Filter.RangerImpurity are competitive to the baseline FS method.

Setting B has the same number of candidate features as Setting A, but only 3 relevant ones. The budget restricts only the one most expensive feature from entering the model. Hence, a good feature selection algorithm primarily needs to separate informative features from noise features here. The boxplots for this setting are either very short or stretched for all methods. This can be explained by the small number of relevant features. The performance almost only depends on whether none, one, or both possible relevant features are included by the algorithm. The genetic algorithm and cFS together with cFS.mean solve this situation for nearly all 100 data sets perfectly (i.e. including exactly the two relevant features). FS and Filter.tTest are slightly behind. The remaining filter methods again find the optimal solution in less cases. Yet overall, every method is able to perfectly solve the given setting in more than two out of three cases.

Setting C (300 candidate features) describes a realistic data scenario in found for example in metabolomic applications [18]. One characteristic of this setting is the strong budget constraint allowing only $\frac {1}{3}$ of the 30 relevant features to enter the model. This configuration therefore combines the challenges of Setting A and B by incorporating many noise features together with the need for an efficient cost strategy. Here the cFS algorithm shows a clear advantage compared to its counterpart FS. Mean trade-off adjustment of the cFS cFS.mean resulted in lower performance than the unadjusted version cFS, with a mean AUC difference of 0.017. The genetic algorithm performs only slightly worse than cFS. The lowest result is obtained with the filter methods, with no great differences between them. Their AUC is similar as for FS, yet, their boxes lie fully below the respective boxes of every other method.

Setting D is in multiple aspects similar to Setting B. However, the main problem is strongly exacerbated by only incorporating information in every 100th feature. Furthermore, the effect size was halved. Nevertheless, the results mainly resemble the ones observed in Setting B. The overall differences between the methods appear smaller, which may be traced back to the smaller amount of information and lower performance maximum.

The Settings E and F have multiple peculiarities. First of all, with *p*=1500 the total number of features is chosen in a high-dimensional area. Non-filter methods are often prone to run-time or convergence problems in these situations. In analogy to Setting D, only around 1 in every 100 features is simulated to carry relevant information. For Setting F, *γ* is set to $\frac {1}{2}$. This results in a similar model size constraint as in Settings A and C, hence enabling an assessment of the impact of adding noise features to the data. On the contrary, Setting E introduces a very special scenario with its value for *γ*. The budget limit is set higher than necessary to include the information of all relevant features. This contradicts the idea of an actual cost constraint, yet can illustrate the properties of the analyzed algorithms in unbounded situations.

While the cFS algorithm is performing best in most other settings, for Setting E it performs worst with its full box below the one of the second lowest performing method. The non-existing constraint on the total relevant information plays into the hands of the FS method. However, a trade-off adaptation can compensate this weakness of cFS, and cFS.mean ranks indeed only slightly behind FS. cGA also performs similarly, seemingly unaffected from the missing information limit. However, the filter methods dominate this setting. Especially Filter.tTest and Filter.RangerImpurity consist only of AUC values above the boxes of all other methods. Only Filter.PraznikJMIM cannot exceed the range of the other methods.

For Setting F, most methods perform very similar. Only models of Filter.PraznikJMIM are notably worse. Both, cFS and FS result in lower AUC values than the remaining non-filter methods. Here, for the first time, the trade-off adaptation cFS.mean outperforms both unadjusted versions (cFS and FS) and shows the best result among all methods.

A clear advantage of the proposed methods compared to the analyzed baseline approaches can be found in the plot of Setting G. In this setting, a non-identity covariance matrix is used and the data generation framework is altered. The budget limits the original features to only include 10 out of 30 relevant ones. However, this limitation could be bypassed here by switching to cheaper surrogate features with high correlation to relevant ones. Hence, the green bar shown in Fig. 3 cannot be interpreted as the optimal result for Setting G. Overall, all non-baseline methods perform similarly well with slight advantages for the cFS algorithm. The trade-off adaptation on average leads to a small decrease in AUC. Notably worse results are obtained with the filter methods, for which all boxplots lie completely under the full boxplot of the cFS approach.

Setting H analyzes the influence of a dependency between effects and costs. Compared to the analogous Setting C, besides a small reduction in AUC differences between the methods, most approaches are unaffected by this adaptation. The typically observed AUC reduction when using cFS.mean compared to cFS is negligibly small here.

Setting I introduces different effect sizes for the relevant features. Here, cFS shows the highest performance, followed by cFS.mean and cGA. Again, baseline methods generally result in notably lower AUC values.

Similarily to Setting I, Setting J defines different effect sizes for relevant features. Additionally, there is a correlation between effect size and costs. The conclusions are comparable to those for Setting H. Baseline methods show overall lower performances, but the differences to our proposed methods are only minor.

In the final Setting K features are no longer normally distributed. Instead we use a more complex distribution with heavy tails and more extreme values. The conclusions are similar to those for previous settings. cFS again shows the highest AUC results, followed by cFS.mean and cGA. All baseline methods perform similarily bad.

#### Run-Time

The run-time is analyzed for all methods and settings. Overall, main influential factors between settings are the dimension of the feature space and the maximum number of relevant features that can be added for the given budget.

In general, the genetic algorithms result in longer run-times than any other method. The only exception to this occurs in Setting E, where cFS takes longer than fGA. As in a greedy forward selection approach, the number of evaluations per iteration scales with the feature dimension, the complexity for applying such a method may become prohibitive in situations with very large *p*. Though evaluating a smaller number of iterations, cGA has in general slightly longer run-times than fGA. This does not necessarily imply a more efficient search strategy of fGA, as the performance of cGA was often slightly higher as well. Similar reasoning can be applied to the greedy forward selection approaches. While not adding computational complexity, cFS shows up to two-fold run-times compared to FS. Applying a trade-off adaptation slightly reduces the run-time compared to cFS. All observed differences in run-time become more severe with higher dimension of the input feature space. The average run-time for filters is negligibly small and dominates all other methods. A detailed overview of the exact median run-times for all methods and simulation settings is tabulated in Additional file 1.

#### Model Size and Detection of Relevant Features

**Fig. 4:**
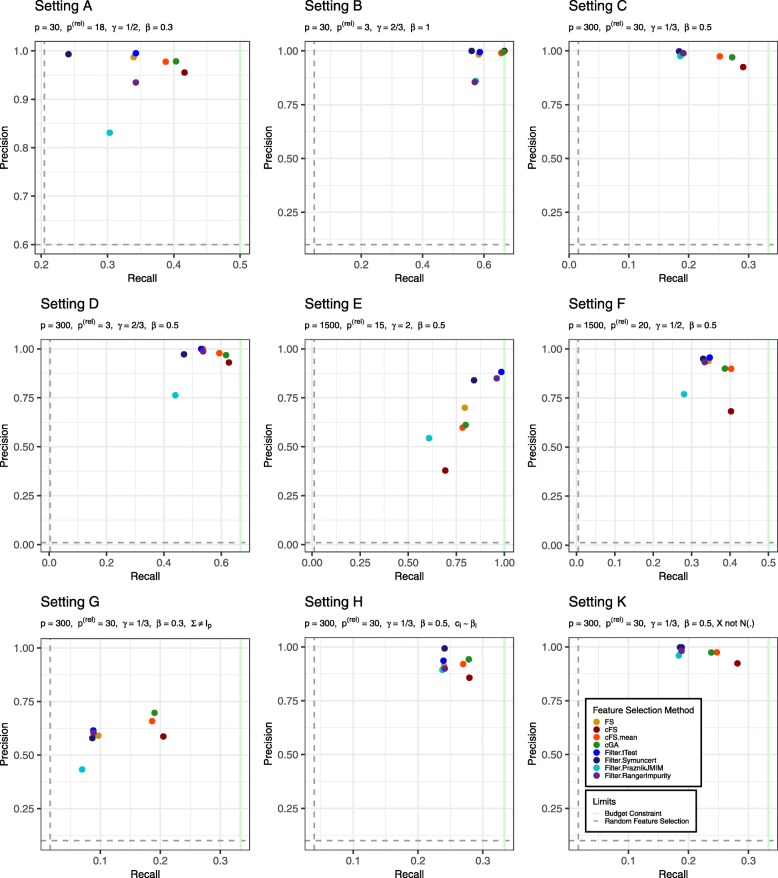
Precision-recall plot comparing analyzed feature selection methods for all simulation settings. Precision corresponds to the ratio of relevant detected features divided by total amount of features in the model. Recall shows the ratio of relevant detected features divided by the total existing number of relevant features. The cost budget defines an upper limit for the recall in the simulations. It is highlighted by a green line. To assess the quality of the feature selection methods precision and recall for selecting features randomly is added to the plots as horizontal and vertical dashed lines. The plot boundaries are re-scaled to depict the area of interest between randomness and optimal values

The final aspect of our simulation analysis concerns the composition of the resulting feature combinations regarding relevant and noise features. To formulate this, we analyze the 2×2 contingency table generated by the variables ’*Is the feature truly relevant?*’ and ’*Was the feature selected?*’. For our 100 data sets, we can then compute the *precision* as the ratio of selected relevant features among the total number of selected features. (→ What portion of the selected features is actually relevant?) This measure focuses on the composition of the selected combination solely. Hence a method selecting only 1 out of 1000 relevant features, but no noise feature would still optimize this criterion. To also focus on the total information available in the feature space we can compute the *recall* as the ratio of selected relevant features among all existing relevant features. (→ What portion of all relevant features was selected?) In settings with a true budget constraint (*γ*<1), this value has an upper limit. With the definition of *c*_max_ according to (7), this limit is *γ*. An illustration of precision and recall for the analyzed methods for simulation Settings A-H and K is given in Fig. 4. The exact values are furthermore tabulated in Table 3.

**Table 3:** Overview of precision and recall of all analyzed feature selection methods for different simulation settings

	Recall									Precision								
	A	B	C	D	E	F	G	H	K	A	B	C	D	E	F	G	H	K
Methods																		
FS	33.9	58.3	19.2	53.7	79.4	34.4	9.7	24.0	18.9	98.7	98.3	98.8	100.0	69.9	93.9	59.1	91.2	98.1
cFS	41.6	66.7	29.1	62.7	69.3	40.2	20.5	27.9	28.2	95.5	100.0	92.5	93.1	37.9	68.2	58.7	85.7	92.3
cFS.mean	38.8	65.7	25.2	59.3	78.3	40.3	18.6	27.0	24.7	97.8	99.0	97.4	97.8	59.8	89.9	65.8	92.0	97.5
cFS.max	37.6	64.0	23.6	57.0	79.1	38.7	16.3	26.3	22.9	98.7	99.0	98.6	98.8	63.5	91.1	65.6	93.0	97.9
fGA	40.6	66.3	27.4	55.0	81.7	38.0	19.0	27.8	23.8	97.9	99.5	98.1	87.3	59.3	83.7	68.1	93.8	98.3
cGA	40.3	66.3	27.3	61.7	79.9	38.6	19.0	27.8	23.8	97.8	99.5	97.0	96.9	61.2	90.0	69.7	94.2	97.4
Filter.tTest	34.3	58.7	18.4	53.0	98.4	34.7	8.8	23.9	18.9	99.5	99.4	99.8	100.0	88.2	95.6	61.5	93.6	99.8
Filter.Symuncert	24.1	56.0	18.4	47.0	84.2	33.0	8.7	24.1	18.6	99.3	100.0	99.6	97.2	84.0	95.0	57.9	99.3	99.8
Filter.PraznikJMIM	30.3	57.3	18.6	44.0	60.8	28.1	7.0	23.8	18.4	83.1	86.0	97.6	76.3	54.4	77.0	43.3	89.3	96.0
Filter.RangerImpurity	34.3	57.0	19.1	53.7	96.0	33.4	8.9	24.1	18.8	93.5	85.5	99.0	98.8	85.0	93.3	60.5	89.9	98.3
Reference																		
Budget constraint	50.0	66.7	33.3	66.7	100.0	50.0	33.3	33.3	33.3	100.0	100.0	100.0	100.0	100.0	100.0	100.0	100.0	100.0
Random selection	20.5	4.7	1.5	0.3	1.1	0.5	1.6	4.0	1.6	60.0	10.0	10.0	1.0	1.0	1.3	10.0	10.0	10.0

Values are given in percent. The results are an extended version of the data shown in Fig. 4. Please refer to the description of this figure for further details

Values of these measures obtained with a *random* feature selection are added as reference. To compute these measures an estimation of the selected relevant features under random selection is needed. Assume a selected feature subset of size *n*. The expected number of relevant features in this subset equals the expected value of the corresponding hypergeometric distribution: $X \sim H(p, p^{(\text {rel})}, n) \Rightarrow E(X) = n\frac {p^{(\text {rel})}}{p}$. We can now derive an estimate for random precision by dividing this expected value by the total number of selected features *n*. The result is independent of the size of the subset and is given by $\frac {p^{(\text {rel})}}{p}$. For recall, *n* does not cancel out. Hence we need to estimate it as the average model size a budget allows: $\frac {c_{\max }}{\text {mean}_{i}(c_{i})}$. We can then estimate the expected value of the hypergeometric distribution by $\frac {c_{\max }}{\text {mean}_{i}(c_{i})}\frac {p^{(\text {rel})}}{p}$. Dividing this number by the total count of relevant features (*p*^(rel)^) gives the random recall value plotted in Fig. 4 and reported in Table 3.

For all settings with a true budget constraint we observe that the new proposed methods cFS, cFS.mean and cGA clearly dominate with respect to recall. However, for precision, in several settings the baseline methods seem to give better results. In particular, cFS always has the highest recall values but lower precision values, compared to cFS.mean and cGA.

In Setting G, a non-identity covariance matrix was used. Hence, the number of selected relevant features does not directly correspond to the amount of detected information.

Setting E - with no true budget constraint on informative features - represents another peculiar situation. Filter methods (except for Filter.PraznikJMIM) dominate this setting with respect to both precision and recall. Among the non-filter methods, the missing restrictive budget to control the overall model size is exploited the most by the cFS algorithm. It often favors very cheap noise features with small random explanatory value over expensive but truly relevant features and hence in general tends to include more features than all other methods. This results in a very low precision value of only 0.379. The trade-off adaptation attenuates this characteristic and leads to precision and recall values close to cGA. As this observation can also be made at Setting F, the adaptation on the cFS shows to be a relevant and necessary extension to the cFS algorithm when dealing with high dimensional data.

**Fig. 5:**
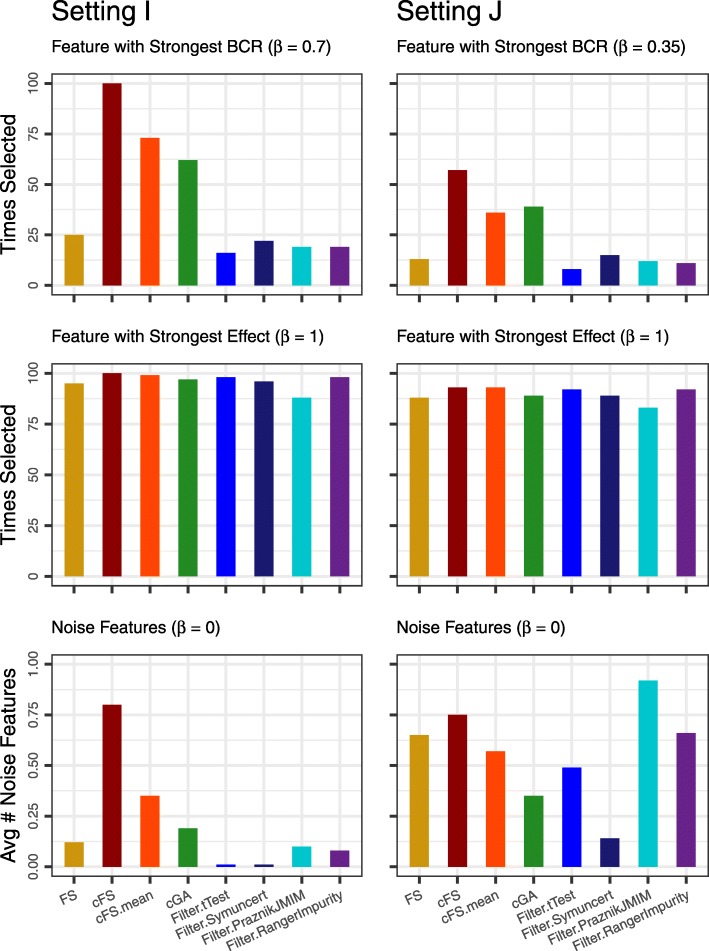
Selection frequency the feature with strongest benefit-cost-ratio (first row) and the feature with strongest effect size (second row). The third row shows the average number of noise features across the simulations

For Settings I and J the precision-recall analysis is not carried out. As these settings include features with different effect sizes, the pure number of detected features is not a suitable measure. Alternatively we inspect the frequency of selection for each feature individually. Plots for every feature are given in Additional file 7. A more concise version with focus on the feature with highest effect, the feature with highest cFS, and the selection of noise features is given in Fig. 5.

In both settings, the feature with strongest effect size is selected almost always by any method. The feature with optimal cFS is more often selected by our proposed methods. It is noteworthy that by design, strong cFS value differences are less frquent for Setting J, due to the correlation between cost and effect size. The number of good cFS candidates (high performance, low cost) is smaller. Hence, the advantage of being able to detect such features is reduced. This explains the smaller performance differences for Setting J compared to Setting I. The plot of noise features leads to similar conclusions as before.

### Real-World Data Simulation Results

**Fig. 6:**
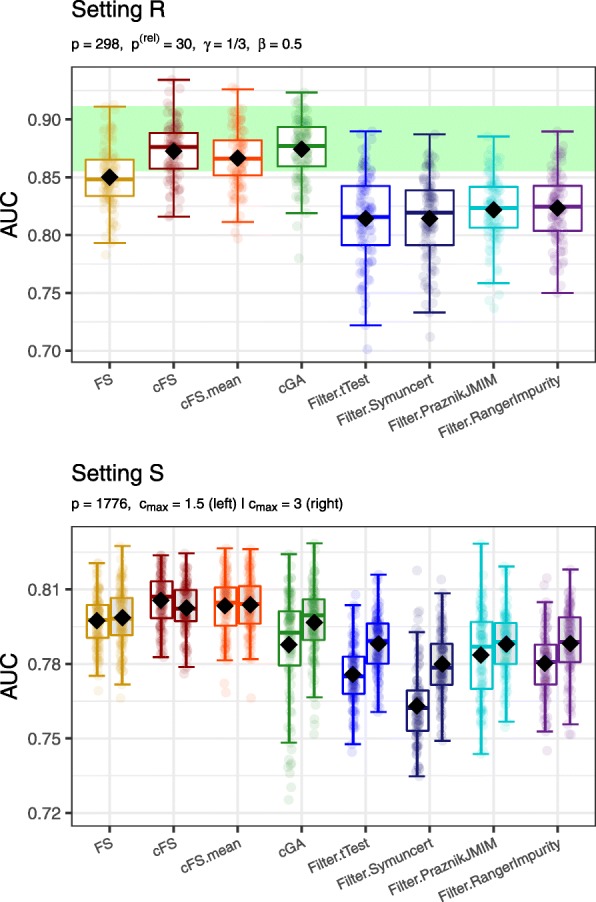
Performance results for the plasmode simulation Setting R and the real world data Setting S. Boxplots for every feature selection method illustrate the distribution of the AUC values obtained for the 100 training-test splits (transparent dots). The black diamonds depict the mean AUC values. A green bar in the top plot highlights the area between the 0.05 and 0.95 quantile of AUC values when always selecting the optimal subset of relevant features that fit in the budget. For Setting S, in the bottom plot, the left elements show the results with *c*_max_=1.5 and the right elements show the results with *c*_max_=3

Figure 6 illustrates boxplots of every analyzed feature selection method for the plasmode Setting R and the real-world Setting S. For Setting R, the plot structure is analogous to the one in Fig. 3. For Setting S, the left elements show the results at *c*_max_=1.5 and the right elements at *c*_max_=3.

The results of Setting R resemble in multiple aspects the results of Setting C. The genetic algorithm and the cFS method perform best. The cFS trade-off adaptation cFS.mean ranks only shortly behind. Again, FS shows better performance than any filter approach, but cannot reach the predictive performance of the three new methods. Similarly to Setting G, no method could reach overall higher AUC values than the model containing all relevant features fitting the budget (green bar of top plot in Fig. 6). Cheaper surrogates of relevant features could have allowed to exceed this performance value, but here no such surrogate structure was found. For Setting S, cFS and its adaptations perform best, followed by the unadapted FS. The genetic algorithms cannot quite hold up with these results. The reason might be the high-dimensional nature of the data set and thus slower convergence of the algorithm. Comparing the two budget limits, no forward selection algorithm can improve its performance with a higher *c*_max_ value. cFS even shows a minor decrease in AUC. In contrast, the genetic algorithms and the filter methods can still improve their performance at *c*_max_=3, but do still not outperform cFS.

With respect to run-time, the results of Setting R are comparable to Setting G. Filter methods resulted in run-times around one second, while the greedy forward selection algorithms required approximately 30 s. The highest run-times were observed for the genetic algorithm with a median value of around 3.5 min. For Setting S, run-times in the range of those for the high-dimensional Setting E were observed. Increasing the budget more than doubled the run-times of non-filter methods, whereas run-times for filter methods did not increase. A detailed overview of the exact values for all methods is tabulated in Additional file 1.

**Fig. 7:**
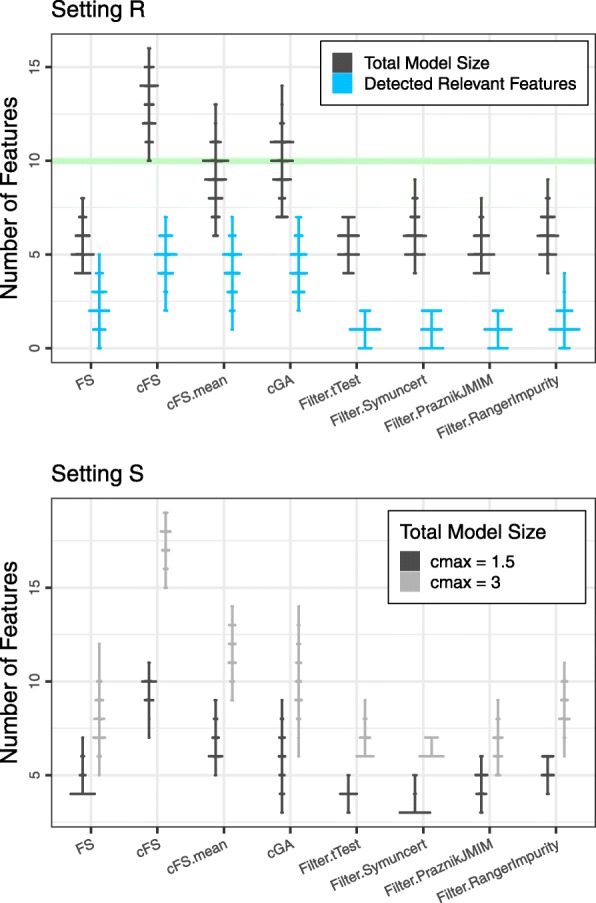
Discretized violin plots of the relevant feature count distribution (blue) and the total model size distribution (black) for the 100 analyzed training-test splits of the plasmode simulation. The green bar indicates the maximum number of relevant features that can be added within the budget of this setting. Bottom: Setting S. Discretized violin plots of the distribution of total model size for the analyzed budget limits *c*_max_=1.5 (black) and *c*_max_=3 (gray)

As final analysis we assess the composition of the resulting feature subsets. We do not compute precision and recall here, as these measures are not valid performance estimates in settings with correlated features and may be misleading. Moreover, these measures cannot be computed for our Setting S in general. Instead we depict the resulting model sizes for every method. For Setting R, we additionally show the number of detected relevant features. In Fig. 7 the distributions of these measures are illustrated using discretized versions of violin plots. Similar to the previous analyses, we indicate the maximum number of relevant features fitting in the defined budget for Setting R, but again comment that reaching an optimum for this measure does not necessarily correspond to an optimal solution. Also, the size of a model by itself does not imply any negative aspect. Statements on model quality are only possible in combination with performance results.

The discrete violin plots of Setting R show, that the FS algorithm as well as all filter methods tend to select smaller feature combinations. Together with the AUC results, one conclusion is that these algorithms in general select too expensive features and reach their budget limit with less overall information than the cost-adapted methods. On the contrary, the cFS method selects rather large feature combinations, yet does not outperform the genetic algorithms. The number of relevant features approximately matches the overall model size, with the exception of cFS, which selects larger models without a tendency to more relevant features. The additional features do not improve, but also not impair the overall performance.

For Setting S, the comparison of model sizes between the methods leads to similar results as before. When increasing the budget, we observe larger models for all methods. The clearly largest increase is found for cFS. As the performance of this method decreases with larger budget, it can be assumed that in this case at least some non-relevant features are selected.

## Discussion

Simulation studies have demonstrated that in scenarios with a “true” budget constraint (i.e. not all available information can be added) cFS, its adaptations and cGA all have better prediction performances compared to the classical FS and to all analyzed filter methods. cFS turned out to have weaknesses for the unbounded Setting E. However, the trade-off adaptation of cFS could overcome this problem and achieved good results in all settings, with at a short run-time. The most versatile approach was the genetic algorithm. It ranked best in multiple different settings and did not show sensitivity to any data composition. However, this comes at the price of an at least five-fold increased run-time compared to cFS.

The interpretation of the precision-recall plots corresponds to the interpretation of the AUC results in multiple aspects. Nevertheless it is important to note, that these measures can not be valued equally for the given task. While reducing the number of noise features in the model is a desirable property, it is more important to include the relevant features. This is reinforced by the clear performance advantage observed for the methods optimizing recall. cFS demonstrates this most clearly. It tends to select relatively large feature subsets including many useful ones, but also multiple noise features. When applying the mean adaptation to this method (cFS.mean), the precision increases while the recall decreases. cGA often ranks in between these methods with both, large precision and recall values.

The results obtained in the real-world settings reinforce the overall findings of the simulations with artificial data. For larger budgets cFS tends to include a higher number of noise features compared to the other methods. Still, in the analyzed scenarios its performance is superior compared to methods without this issue. For higher budgets, the effects of this problem might become more important.

## Conclusion

In this paper, we addressed the problem of selecting an optimal feature subset in the presence of individual feature costs and a hard budget constraint. This topic is crucial in many practical applications, but only little research is devoted to it up to date. We proposed three new methods, cFS, fGA and cGA, that extend well known and often applied feature selection algorithms to also handle cost constraints. For cFS, a general trade-off adaptation strategy of the introduced benefit-cost ratio was proposed and two explicit options for this strategy were discussed. All proposed methods share the advantage of not requiring cost-related hyperparameters. Therefore, no additional tuning steps are necessary to translate a standard type algorithm into one with the proposed cost-conscious extensions.

Altogether, we recommend to use cGA in situations without extreme computational resource constraints for its robustness in a wide variety of data settings and its generalized implementation allowing to define a completely unconstrained fitness function. If run-time is an essential factor, then the cFS.mean method also performs well in many data scenarios, since it results in overall only slightly lower AUC values compared to cGA, while still being rather robust to large budgets and selection of noise features. In almost all settings with a clear budget constraint, the unadapted cFS provides the best results. However, because of its clear inferiority in Setting E and its tendency to add noise features, we do not recommend this method as our first choice. Finally, we would advise to check if an actual budget constraint is given in advance. If the actual budget is not limited, an unnecessary adaptation of feature selection methods may strongly impair the final results.

Beyond the scope of this work, many extensions of the current methods are possible. Currently, the selection of a feature subset is performed according to the AIC. Other performance measures evaluated in a cross-validation setting could further improve the general predictive performance. Moreover, this would broaden the field of applicable modelling methods, which is currently only limited by the applicability of the AIC. In principle, each of the proposed methods could be extended to many supervised learning tasks. Finally, analogous adaptations on many other feature selection approaches are possible to further extend the spectrum of available methods in this field.

## Supplementary information

**Additional file 1** **Run-time overview table**. Median run-time in seconds for the execution of one feature selection run. All results of methods and Settings analyzed in the simulations of this paper are given rounded to two digits.

**Additional file 2** **Extended version of Fig. **3. Performance results for simulation Settings A to K. Boxplots for every feature selection method illustrate the distribution of the AUC values obtained for the 100 data sets (transparent dots). The black diamonds depict the mean AUC values. A horizontal bar highlights the area between the 0.05 and 0.95 quantile of AUC values when always selecting the cheapest subset (green) or the best real cFS subset (golden) of relevant features that fit in the budget. Both correspond to a univariately optimal solution.

**Additional file 3** **Extended version of Fig. **4 without re-scaling. Precision-recall plot comparing all analyzed feature selection methods for the main simulation settings. Precision corresponds to the ratio of relevant detected features divided by the total number of features in the model. Recall shows the ratio of relevant detected features divided by the total number of truly relevant features. The cost budget defines an upper limit for the recall in the simulations. It is indicated by a green line. To assess the quality of the feature selection methods, values for precision and recall of selecting features randomly are added to the plots as horizontal and vertical dashed lines.

**Additional file 4** **Extended version of Fig. **6. Performance results for the plasmode simulation Setting R and the real world data Setting S. Boxplots for every feature selection method illustrate the distribution of the AUC values obtained for the 100 training-test splits (transparent dots). The black diamonds depict the mean AUC values. A green bar in the top plot indicates the area between the 0.05 and 0.95 quantile of AUC values when always selecting the optimal subset of relevant features that fit in the budget. For Setting S, the left elements show the results with *c*_max_=1.5 and the right elements show the results with *c*_max_=3.

**Additional file 5** **Extended version of Fig. **7. Top: Setting R. Discretized violin plots of the relevant feature count distribution (blue) and the total model size distribution (black) for the 100 analyzed training-test splits of the plasmode simulation. The green bar indicates the maximum number of relevant features that can be added within the budget of this setting. Bottom: Setting S. Discretized violin plots of the total model size distribution for the analyzed budget limits *c*_max_=1.5 (black) and *c*_max_=3 (gray).

**Additional file 6** **Screening adequate size of*c***_**max**_** for Setting S**. Plot of AUC values of all filter methods for different values of *c*_max_. After approximately *c*_max_=3 no improvement for larger budgets is assumed.

**Additional file 7** **Complete version of Fig. **5. Individual barplots for every relevant feature of Settings I and J. The y-axis shows the frequency of selection for every analyzed method.

## Data Availability

The datasets used and/or analysed during the current study are available from the corresponding author on reasonable request.

## Abbreviations

AIC: Akaike information criterion
AUC: Area under the receiver operating characteristic curve
BCR: Benefit-cost ratio
cFS: Cost-constrained forward selection
cGA: Cost-preserving genetic algorithm
DFG: Deutsche Forschungsgemeinschaft
fGA: Genetic algorithm with fitness adaptation
FS: Forward selection with naive cost limitation
NMR: Nuclear magnetic resonance

## Footnotes

1 A total of 20 features each have a 50% chance of entering the model. The budget is violated if more than 10 are selected, and *X*∼*B*(20,0.5)⇒*P*(*X*>10)≈0.412

2 $P(\mathrm {Adding~a~feature~to~}s) = \frac {\# \mathrm {Features~not~in~}s}{\# \text {Features}} = \frac {490}{500} = 0.98$

3 numares AG, Am BioPark 9, 93053 Regensburg
