# Supplementary material for: Cost-Constrained feature selection in binary classification: adaptations for greedy forward selection and genetic algorithms
Source: BMC Bioinformatics. 2020 Jan 28;21:26. doi: 10.1186/s12859-020-3361-9 (PMC6986087; doi:10.1186/s12859-020-3361-9)
Supplement: Supplementary file 2 — Additional file 2 Extended version of Fig. 3. Performance results for simulation Settings A to K. Boxplots for every feature selection method illustrate the distribution of the AUC values obtained for the 100 data sets (transparent dots). The black diamonds depict the mean AUC values. A horizontal bar highlights the area between the 0.05 and 0.95 quantile of AUC values when always selecting the cheapest subset (green) or the best real cFS subset (golden) of relevant features that fit in the budget. Both correspond to a univariately optimal solution. [file 12859_2020_3361_MOESM2_ESM.pdf]

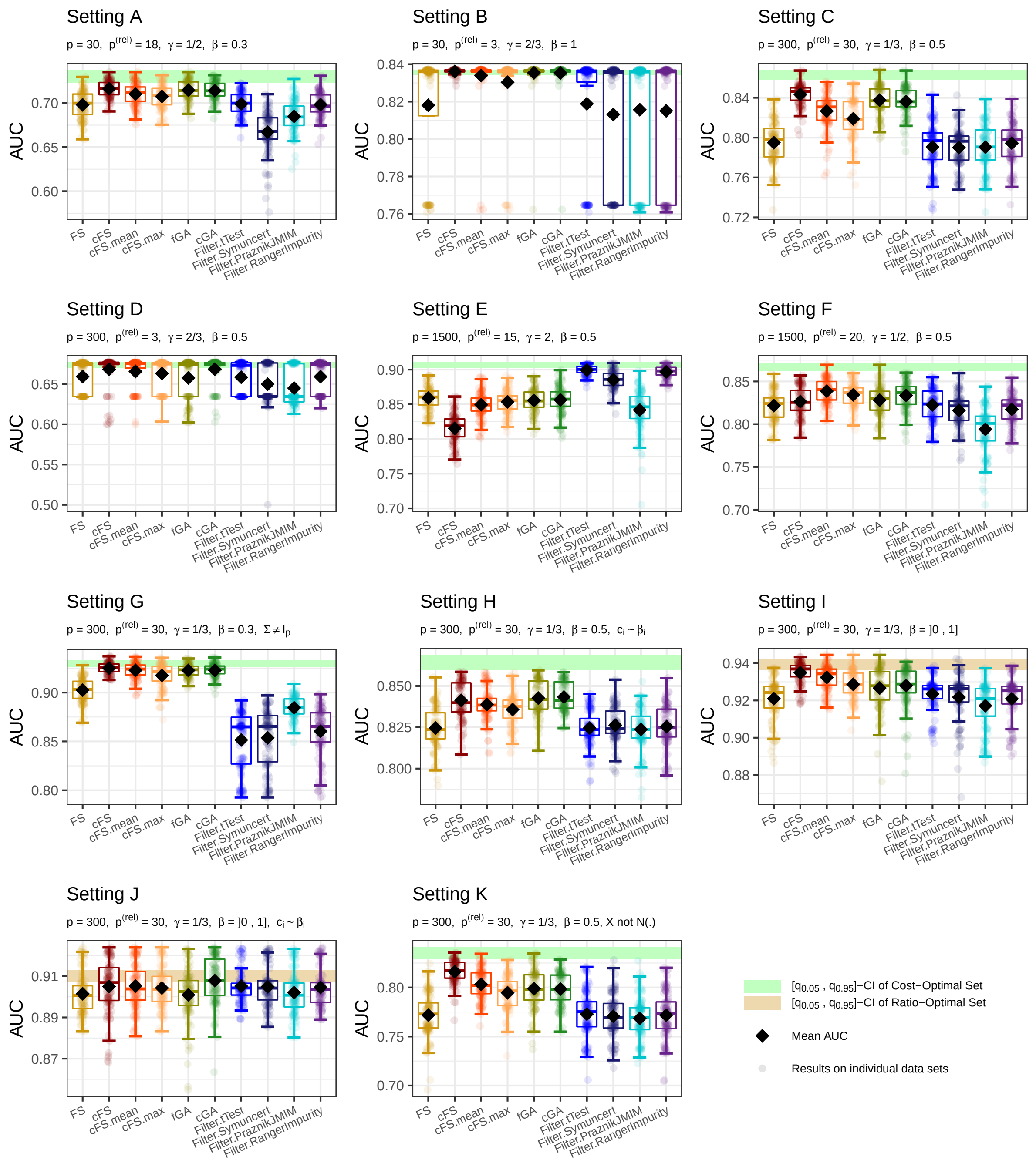

Setting A
p = 30, p(rel) = 18, γ = 1/2, β = 0.3
Setting B
p = 30, p(rel) = 3, γ = 2/3, β = 1
Setting C
p = 300, p(rel) = 30, γ = 1/3, β = 0.5
Setting D
p = 300, p(rel) = 3, γ = 2/3, β = 0.5
Setting E
p = 1500, p(rel) = 15, γ = 2, β = 0.5
Setting F
p = 1500, p(rel) = 20, γ = 1/2, β = 0.5
Setting G
p = 300, p(rel) = 30, γ = 1/3, β = 0.3, Σ ≠ Ip
Setting H
p = 300, p(rel) = 30, γ = 1/3, β = 0.5, ci ~ βi
Setting I
p = 300, p(rel) = 30, γ = 1/3, β =]0 , 1]
Setting J
p = 300, p(rel) = 30, γ = 1/3, β =]0 , 1], ci ~ βi
Setting K
p = 300, p(rel) = 30, γ = 1/3, β = 0.5, X not N(.)
AUC
FS
cFS
cFS.mean
cFS.max
fGA
cGA
Filter.tTest
Filter.Symuncert
Filter.PraznikJMIM
Filter.RangerImpurity
[q0.05 , q0.95]−CI of Cost−Optimal Set
[q0.05 , q0.95]−CI of Ratio−Optimal Set
Mean AUC
Results on individual data sets
