# Supplementary material for: Cost-Constrained feature selection in binary classification: adaptations for greedy forward selection and genetic algorithms
Source: BMC Bioinformatics. 2020 Jan 28;21:26. doi: 10.1186/s12859-020-3361-9 (PMC6986087; doi:10.1186/s12859-020-3361-9)
Supplement: Supplementary file 3 — Additional file 3 Extended version of Fig. 4 without re-scaling. Precision-recall plot comparing all analyzed feature selection methods for the main simulation settings. Precision corresponds to the ratio of relevant detected features divided by the total number of features in the model. Recall shows the ratio of relevant detected features divided by the total number of truly relevant features. The cost budget defines an upper limit for the recall in the simulations. It is indicated by a green line. To assess the quality of the feature selection methods, values for precision and recall of selecting features randomly are added to the plots as horizontal and vertical dashed lines. [file 12859_2020_3361_MOESM3_ESM.pdf]

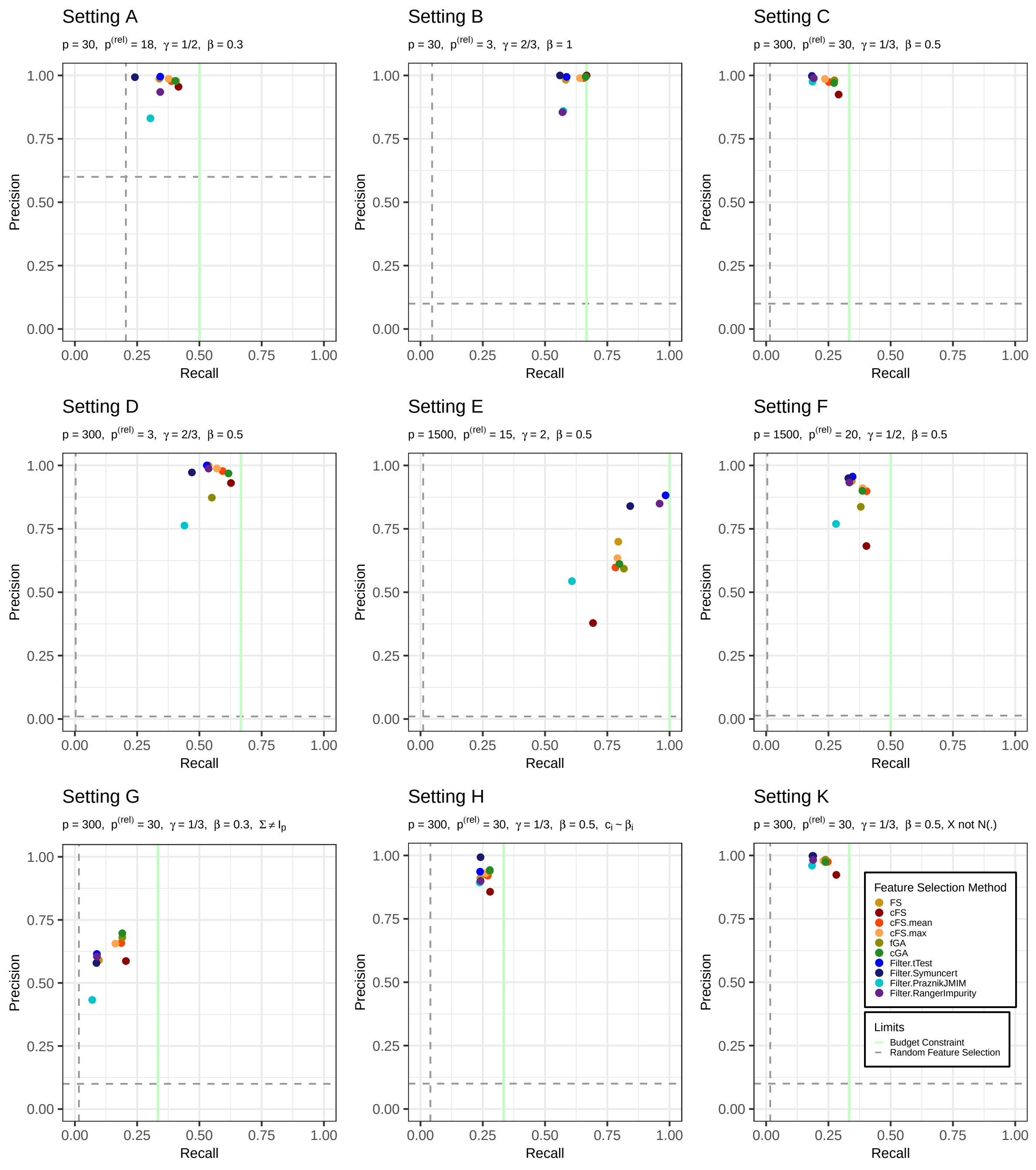
Setting A
p = 30, p^(rel) = 18, γ = 1/2, β = 0.3
Setting B
p = 30, p^(rel) = 3, γ = 2/3, β = 1
Setting C
p = 300, p^(rel) = 30, γ = 1/3, β = 0.5
Setting D
p = 300, p^(rel) = 3, γ = 2/3, β = 0.5
Setting E
p = 1500, p^(rel) = 15, γ = 2, β = 0.5
Setting F
p = 1500, p^(rel) = 20, γ = 1/2, β = 0.5
Setting G
p = 300, p^(rel) = 30, γ = 1/3, β = 0.3, Σ ≠ I_p
Setting H
p = 300, p^(rel) = 30, γ = 1/3, β = 0.5, c_i ~ β_i
Setting K
p = 300, p^(rel) = 30, γ = 1/3, β = 0.5, X not N(.)
Precision
Recall
0.00
0.25
0.50
0.75
1.00
Feature Selection Method
FS
cFS
cFS.mean
cFS.max
fGA
cGA
Filter.tTest
Filter.Symuncert
Filter.PraznikJMIM
Filter.RangerImpurity
Limits
Budget Constraint
Random Feature Selection
