# Supplementary figures and images for: Cost-Constrained feature selection in binary classification: adaptations for greedy forward selection and genetic algorithms
Source: BMC Bioinformatics. 2020 Jan 28;21:26. doi: 10.1186/s12859-020-3361-9 (PMC6986087; doi:10.1186/s12859-020-3361-9)

## Setting R

$p = 298$ ,  $p^{(rel)} = 30$ ,  $\gamma = 1/3$ ,  $\beta = 0.5$

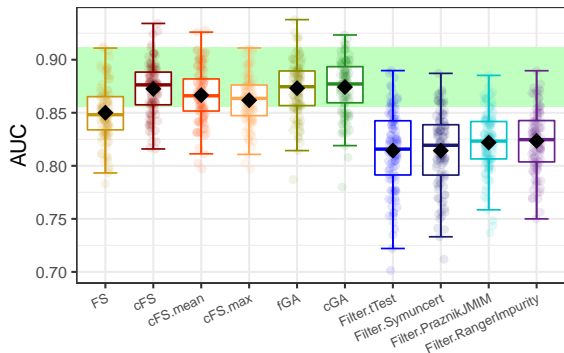

## Setting S

$p = 1776$ ,  $c_{max} = 1.5$  (left) |  $c_{max} = 3$  (right)

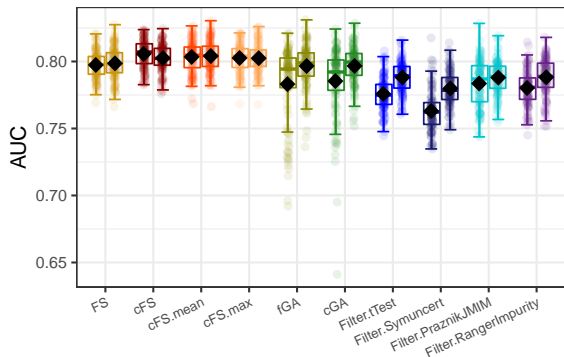

Supplement: Supplementary file 4 — Additional file 4 Extended version of Fig. 6. Performance results for the plasmode simulation Setting R and the real world data Setting S. Boxplots for every feature selection method illustrate the distribution of the AUC values obtained for the 100 training-test splits (transparent dots). The black diamonds depict the mean AUC values. A green bar in the top plot indicates the area between the 0.05 and 0.95 quantile of AUC values when always selecting the optimal subset of relevant features that fit in the budget. For Setting S, the left elements show the results with cmax=1.5 and the right elements show the results with cmax=3. [file 12859_2020_3361_MOESM4_ESM.pdf]

# Setting R

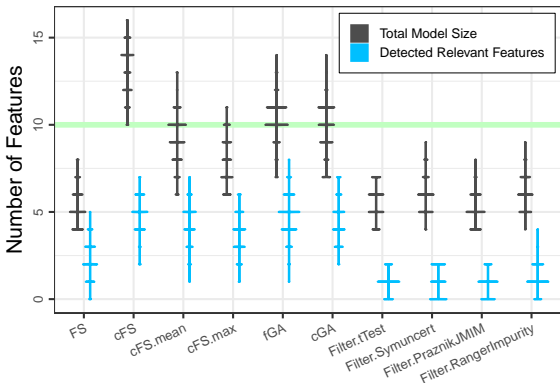

# Setting S

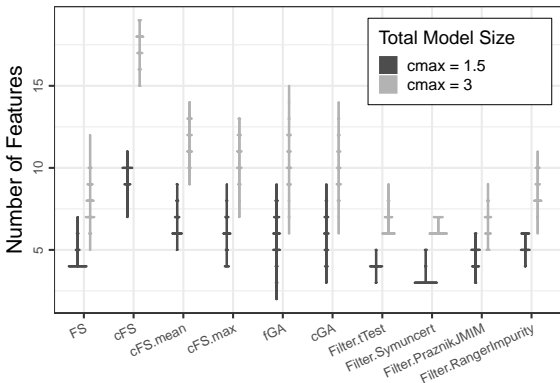

Supplement: Supplementary file 5 — Additional file 5 Extended version of Fig. 7. Top: Setting R. Discretized violin plots of the relevant feature count distribution (blue) and the total model size distribution (black) for the 100 analyzed training-test splits of the plasmode simulation. The green bar indicates the maximum number of relevant features that can be added within the budget of this setting. Bottom: Setting S. Discretized violin plots of the total model size distribution for the analyzed budget limits cmax=1.5 (black) and cmax=3 (gray). [file 12859_2020_3361_MOESM5_ESM.pdf]

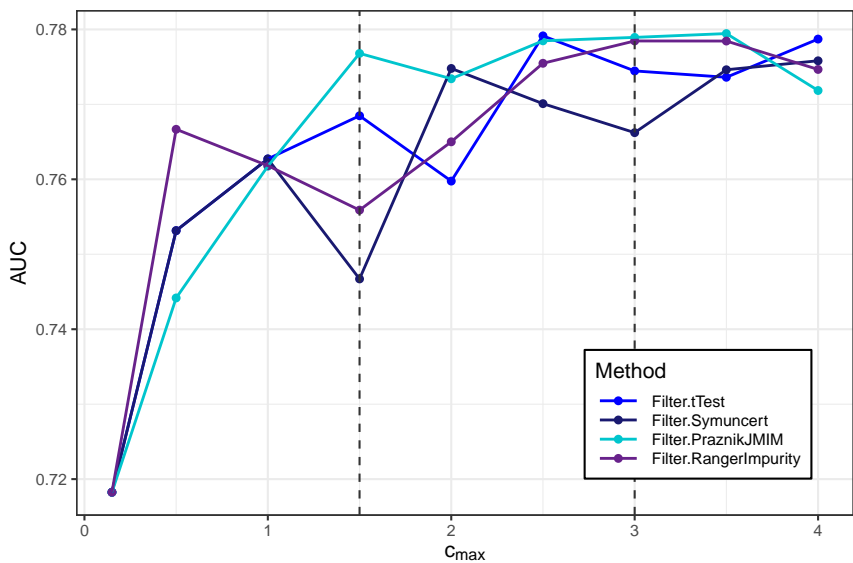

Supplement: Supplementary file 6 — Additional file 6 Screening adequate size ofcmax for Setting S. Plot of AUC values of all filter methods for different values of cmax. After approximately cmax=3 no improvement for larger budgets is assumed. [file 12859_2020_3361_MOESM6_ESM.pdf]

# Setting I

Number of Models the Feature was Selected into

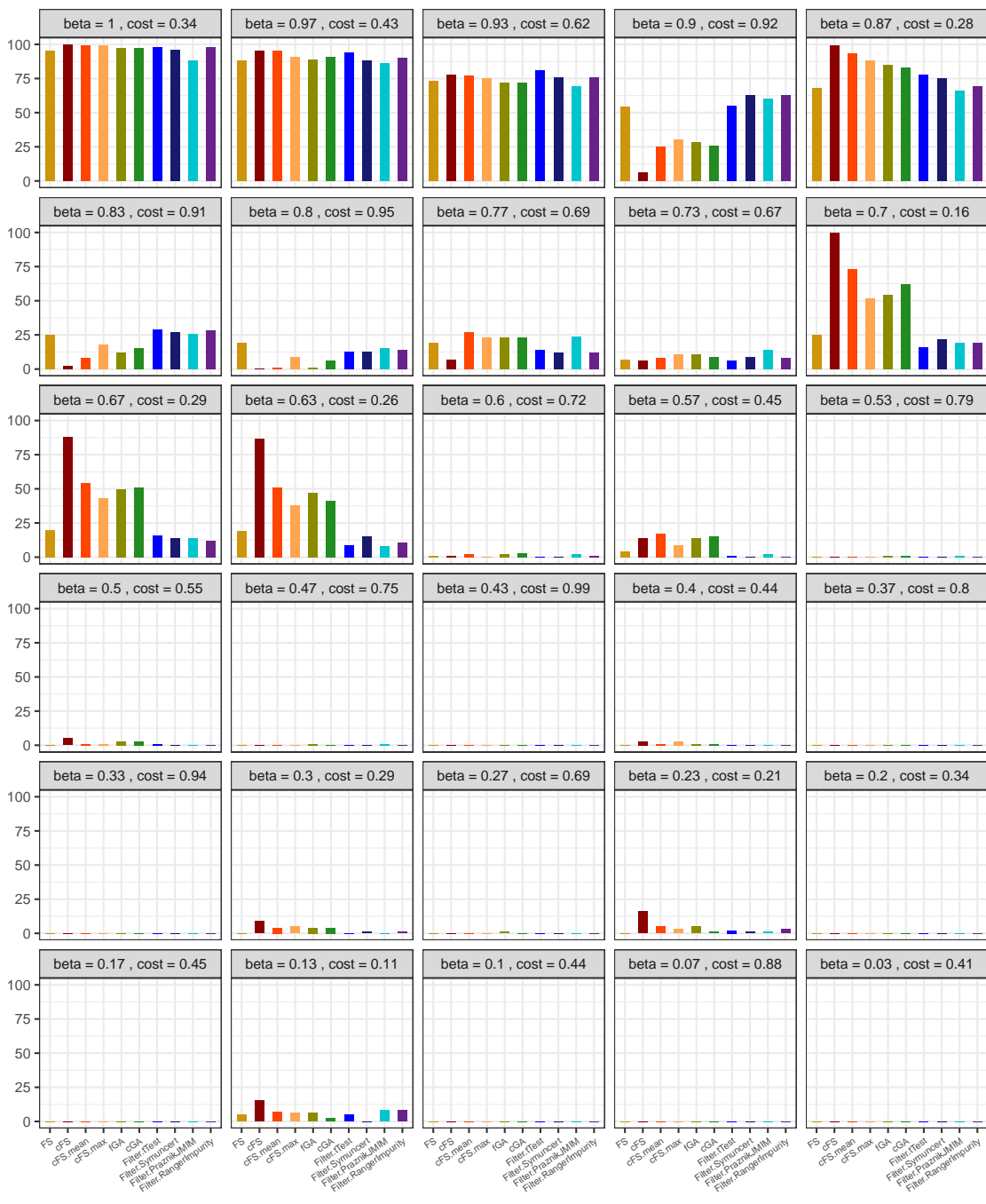

### Setting J

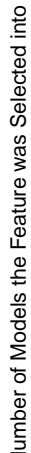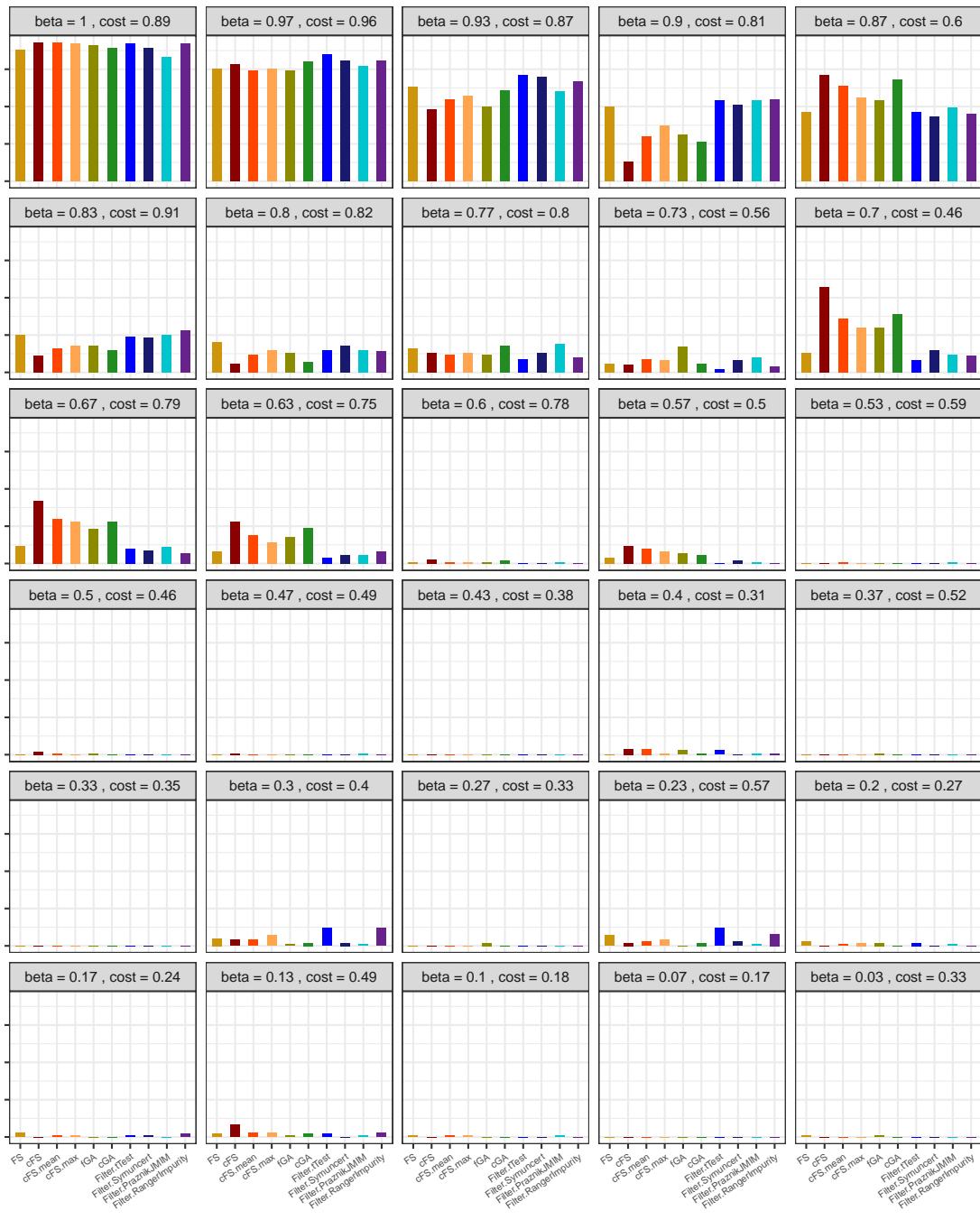

Supplement: Supplementary file 7 — Additional file 7 Complete version of Fig. 5. Individual barplots for every relevant feature of Settings I and J. The y-axis shows the frequency of selection for every analyzed method. [file 12859_2020_3361_MOESM7_ESM.pdf]
